# Sorption and Magnetic Properties of Oxalato-Based Trimetallic Open Framework Stabilized by Charge-Assisted Hydrogen Bonds

**DOI:** 10.3390/ijms23031556

**Published:** 2022-01-29

**Authors:** Tadeusz Mikołaj Muzioł, Natalia Tereba, Robert Podgajny, Robert Pełka, Dominik Czernia, Marek Wiśniewski, Stanisław Koter, Grzegorz Wrzeszcz

**Affiliations:** 1 Faculty of Chemistry, Nicolaus Copernicus University in Toruń, Gagarina 7, 87-100 Toruń, Poland; natalia.tereba@wp.pl (N.T.); Marek.Wisniewski@umk.pl (M.W.); skoter@umk.pl (S.K.); wrzeszcz@chem.umk.pl (G.W.); 2 Faculty of Chemistry, Jagiellonian University, Gronostajowa 2, 30-387 Kraków, Poland; robert.podgajny@uj.edu.pl; 3 Institute of Nuclear Physics of Polish Academy of Sciences, Radzikowskiego 152, 31-342 Kraków, Poland; robert.pelka@ifj.edu.pl (R.P.); dominik.czernia@ifj.edu.pl (D.C.)

**Keywords:** coordination polymer, oxalate complex, single crystal XRD, magnetic properties, sorption properties, charge assisted hydrogen bonds

## Abstract

We report a new structure of {[Co(bpy)_2_(ox)][{Cu_2_(bpy)_2_(ox)}Fe(ox)_3_]}_n_·8.5nH_2_O **NCU-1** presenting a rare ladder topology among oxalate-based coordination polymers with anionic chains composed of alternately arranged [Cu_2_(bpy)_2_(ox)]^2+^ and [Fe(ox)_3_]^3−^ moieties. Along the a axis, they are separated by Co(III) units to give porous material with voids of 963.7 Å^3^ (16.9% of cell volume). The stability of this structure is assured by a network of stacking interactions and charge-assisted C-H…O hydrogen bonds formed between adjacent chains, adjacent cobalt(III) units, and alternately arranged cobalt(III) and chain motifs. The soaking experiment with acetonitrile and bromobenzene showed that water molecules (8.5 water molecules dispersed over 15 positions) are bonded tightly, despite partial occupancy. Water adsorption experiments are described by a D’arcy and Watt model being the sum of Langmuir and Dubinin–Serpinski isotherms. The amount of primary adsorption sites calculated from this model is equal 8.2 mol H_2_O/mol, being very close to the value obtained from the XRD experiments and indicates that water was adsorbed mainly on the primary sites. The antiferromagnetic properties could be only approximately described with the simple Cu^II^-ox-Cu^II^ dimer using **H** = −*J*·**S_1_**·**S_2_**, thus, considering non-trivial topology of the whole Cu-Fe chain, we developed our own general approach, based on the semiclassical model (SC) and molecular field (MF) model, to describe precisely the magnetic superexchange interactions in **NCU-1**. We established that Cu(II)-Cu(II) coupling dominates over multiple Cu(II)-Fe(III) interactions, with *J*_CuCu_ = −275(29) and *J*_CuFe_ = −3.8(1.6) cm^−1^ and discussed the obtained values against the literature data.

## 1. Introduction

Oxalate anions act as bridges assuring the effective transfer of magnetic couplings between paramagnetic centers [1]. The large number of coordination modes defined for this anion [2] enables the tailoring of the formed networks. Therefore, oxalato-based coordination polymers were synthesized and studied as materials showing numerous topologies (chains, layers, or 3D networks) [3,4,5,6,7,8,9,10,11,12,13,14] and hence, also different properties, being e.g., single chain magnets [1,15], water soluble magnets [10,16], chiral magnets [17], spin-crossover compounds [18], luminescent materials [19,20,21], NLO materials [22], photocatalysts and catalysts [23,24], materials showing conductivity [11,25,26], sorption materials [27,28], and precursors for nanomaterials possessing pharmacological potential [29].

Formation of 2D and 3D frameworks can be achieved using different templates (e.g., quaternary amine cations and [M(AA)_3_]^2+^ cations with D_3_ symmetry) [3]. To limit dimensions of the formed compounds, additional auxiliary capping ligands are used [30,31] as well as crown ethers [10]. Ladder chain was detected in [Cu_2_(bpy)_2_(ox)Cr/Fe(ox)_3_]^−^ 1D compounds [4,32] and the interactions were described using 0D model and a single exchange parameter because of the dominating antiferromagnetic interactions in the copper dimer over other couplings. For the description of the magnetic properties of homo- and heterometallic oxalato-based chains, the Heisenberg model was often used with a single exchange parameter [30,33]. A more complex description was proposed for a chain with two copper(II) ions in the asymmetric unit [1]. To properly model χT vs. T curve, the chain was split into ferromagnetic trimers which were subsequently antiferromagnetically coupled. Five exchange parameters were proposed for a trimetallic MnCuCr system showing a highly complex topology with three different ligands acting as bridges [8]. In modeling of the exchange pathways via oxalate anion connecting clusters into a chain, a classical spin approach useful for the description of systems with high spin (in this case S = 5) and relatively weak coupling between them was applied.

Parallel to the above mentioned works of Kanizaj et al. [4,32] we aimed to prepare a trimetallic (Co-Fe-Cu) compound with oxalate anions acting as bridges starting with tris(oxalato)ferrate unit and bpy as a capping ligand. Oxalate anions also offer an opportunity to create a robust hydrogen bond network. Hence, we intended to exploit these properties for the preparation of 1D chains forming 3D network. As the result, we have obtained new oxalato-based trimetallic open framework {[Co(bpy)_2_(ox)][{Cu_2_(bpy)_2_(ox)}Fe(ox)_3_]}_n_·8.5nH_2_O **NCU-1** (NCU—Nicolaus Copernicus University). In **NCU-1**, the [{Cu_2_(bpy)_2_(ox)}Fe(ox)_3_]^−^ 1D oxalate-bridged chains [32] are spontaneously co-crystallized with [Co(bpy)_2_(ox)]^3+^ cations, exploiting charge-assisted hydrogen bonds and π-π interactions, to establish the new coordination secondary building unit (SBU) for rigid porous architectures providing a new generation of magnets with predesigned and controllable structures susceptible for postsynthetic modifications [34]. The global approach for the more complete description of 1D magnetic properties was applied and water sorption/desorption properties were studied in detail, to provide the new quality born by this 1D modular form.

## 2. Results and Discussion

### 2.1. Synthesis

According to our procedure, two products were formed. First, the salmon crystals of **NCU-1** are formed, and then green rods of [Cu(NCS)_2_(bpy)] [35] emerged. Nevertheless, owing to the time gap in crystallization of both complexes, they can be separately collected, and pure {[Co(bpy)_2_(ox)][{Cu_2_(bpy)_2_ox}Fe(ox)_3_]}_n_·8.5nH_2_O can be afforded. The composition of **NCU-1** indicates significant changes to the substrates, beginning with the partial decomposition of [Fe(ox)_3_]^3−^ and dissociation of [Cu(bpy)_2_NCS]^+^. These processes were accompanied by coordination of ox^2−^ to Cu(II), and uptake of both ox^2−^ and bpy by Co(II) centers, the latter followed by oxidation of Co(II) to Co(III) in the air during the crystallization process. Finally, new forms were self-assembled with the remaining [Fe(ox)_3_]^3−^ metaloligands. It should be noted that the final compound was obtained solely according to the procedure described in the experimental part. The starting point was [Cu(bpy)_2_(NCS)](NO_3_). Although, theoretically more appropriate direct strategies failed; an attempt of synthesis from simple salts and bpy or using the building blocks approach with [Cu_2_(bpy)_2_(ox)(H_2_O)_2_] [36] did not yield any salmon crystals. We also tried to use K_3_[Cr(ox)_3_]·3H_2_O but the chromium analog was not synthesized probably because of its inertness.

### 2.2. Structure Description

**Figure 1:**
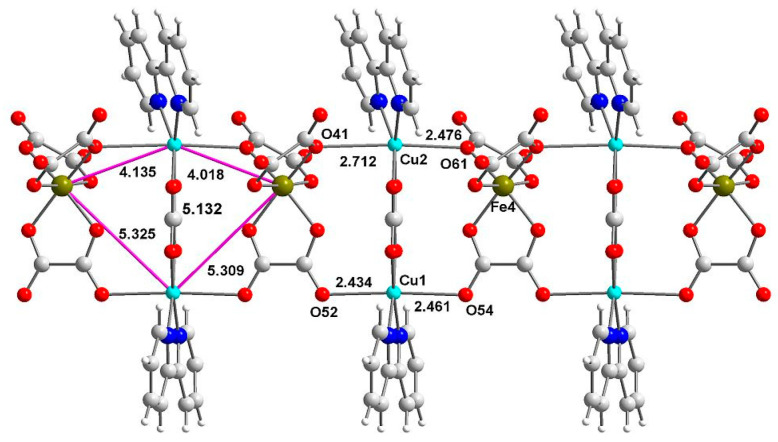
The topology of the anionic [{Cu_2_(bpy)_2_ox}Fe(ox)_3_]^−^ chain is presented with selected bond lengths and intermetallic distances. Cobalt(III) unit and crystallization water molecules are skipped.

{[Co(bpy)_2_(ox)][{Cu_2_(bpy)_2_(ox)}Fe(ox)_3_]}_n_·8.5nH_2_O crystallizes in the monoclinic P2_1_/c space group with all atoms found in the general positions and with the whole formula motif composing the asymmetric unit (Figure S1). There are 8.5 crystallization water molecules dispersed over 15 sites. The structure is composed of anionic chains [{Cu_2_(bpy)_2_(ox)}Fe(ox)_3_]^−^ and complex cations intercalated between them, whereas water molecules are found in large channels (11.6 × 9.0 Å) of the network. The chain is composed of alternately arranged copper dimers and tris(oxalato)ferrate anions connected via bidentate/monodentate and bidentate/bismonodentate oxalate bridges (Figure 1). The bridging oxalate anions are coordinated bidentately to iron(III) cations and monodentately to copper(II) cations either by the outer oxygen atoms (mode 1) or via the inner oxygen atoms (mode 2) (Figure S2). Because of chain topology and manner of coordination binding between tris(oxalato)ferrate blocks and copper(II) dimers, four different Fe-Cu distances (4.018–5.325 Å) are formed. These are the shortest intermetallic distances in this compound and they fall in the range found for structures with identical topology [32]. In the dimer copper(II), cations are coupled via bisbidentately coordinated oxalate anion (mode 3) (Figure S2) with the Cu-Cu distance (5.132 Å) falling in the range found for dimers with two copper(II) ions bridged by bisbidentately coordinated oxalate anion with four short Cu-O_ox_ distances [4,32] (Table S2, Figure S3). This indicates that there are many pathways of possible coupling between paramagnetic metal cations and hence, an extremely complex model of exchange interactions is expected.

Both copper(II) cations show elongated octahedral (S_OC_ = 1.325 for Cu1 and S_OC_ = 2.771 for Cu2) environments, with different deviation from planarity in the equatorial plane (S_SP-4_ = 0.412 for Cu1 and S_SP-4_ = 1.405 for Cu2) [37]. The coordination sphere is composed of two nitrogen atoms from bpy molecules and four oxygen atoms from oxalate anions in the coordination sphere. The equatorial plane is formed by two oxygen atoms (1.9691(19) to 1.9787(19) Å) and two nitrogen atoms (1.965(3) to 1.983(2) Å), whereas oxygen atoms in axial positions form very long Cu-O bonds ranging from 2.434(3) to 2.712(2) Å with the longest value corresponding to a semi-coordination (Table S3). These distances are similar to those found in ammonium and potassium compounds [32] apart from Cu1-O54 and Cu2-O61 which are significantly shorter than the bond lengths found in the ammonium and potassium complexes (Figure S4). In the reported structure, the two equatorial short Cu-O distances are similar and we can anticipate significant antiferromagnetic Cu-Cu couplings, whereas substantial differences in those bonds result in the reduction of the exchange integral value [12]. Iron(III) is found in a trigonally distorted octahedral environment formed by six oxygen atoms from oxalate anions. The Fe-O bonds range from 2.005(2) to 2.023(2) Å and are in the range observed for bridging and terminal oxalate anions [38,39,40]. The cobalt(III) cations coordination sphere consists of two oxygen atoms from bidentately coordinated oxalate anion (mode 4, 1.893(2)–1.900(2) Å) (Figure S2) and four nitrogen atoms coming from bpy (1.947(3)–1.954(3) Å) ligands. The latter bonds fall in the range found for Co-N_ar_ bonds, clearly indicating the low spin configuration by both experimental (XRD) and theoretical methods [41,42,43] and +3 (3.12) oxidation number by bond valence sum calculation [44].

**Figure 2:**
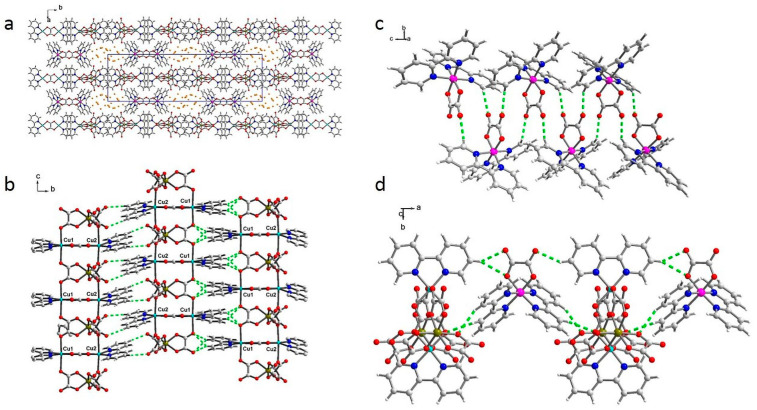
Packing of **NCU-1** along the c axis shows channels filled with crystallization water molecules (in orange) (**a**), interactions between chains along the b axis with marked hydrogen bonds (in green) and stacking interactions formed by N1 and N11 bpy ligands (**b**), interactions of cobalt(III) units along the c axis with marked hydrogen bonds (in green) and a zipper formed by oxalate anions (**c**), and interactions of cobalt(III) units and chains with marked hydrogen bonds (marked in green) running along the a axis (**d**).

Up to the best of our knowledge, such a ladder represents a very rare topology [4,32] with oxalate anions connecting iron(III) and copper(II) and rungs made of bisbidentately coordinated oxalate anions joining both copper(II) cations. Their superposition showed differences in O41/O5 oxalate position and bpy position due to the significant twist observed in **NCU-1** (Figure S5). The already published structures are much more tightly packed due to small ions (potassium or ammonium) connecting adjacent chains either via coordination bonds (potassium) or by hydrogen bonds (ammonium). In these compounds there is only one water molecule (ammonium compound) or the crystallization water molecule is absent (potassium compound). Therefore, for the models with removed solvent molecules, the packing indices are 68.1 and 75.1%, respectively [45]. The voids accessible for solvent are present only for ammonium compound and account for only 1.0% of the cell volume and 16.4 Å^3^ per cell. In the reported structure we exploited the ability of oxalate anions for creation of non-covalent interactions. The packing shows channels running along the c axis filled with crystallization water molecules (Figure 2a). Hence, the additional coordination unit introduces significant changes in symmetry and even more importantly in the network—large and stable channels are formed with cobalt units separating the chains along the a axis. It results in a much lower packing index of the backbone being 61.6% and the volume accessible for the solvent is 963.7 Å^3^ (16.9%) which is sufficient for sorption of gases and/or solvents.

The crucial problem is the stability of such an open framework which is located between a classical covalently bonded 3D coordination MOF and a hydrogen-bonded organic framework (HOF), which relies on weak (mainly hydrogen bonds) interactions [46]. In the reported case, an additional moiety generates voids and we exploited oxalate ability for non-covalent interactions which were able to maintain the permanent porosity [47,48,49]. In **NCU-1**, the covalent bonds are formed only along the chain between copper and iron moieties, whereas in two other directions weak intermolecular interactions (between cobalt units as well as cobalt units and chains) are present. The crystal network is maintained by numerous C-H…O hydrogen bonds with hydrogen atoms coming from bpy molecules and acceptor atoms from all oxalate anions as well as by π-π interactions between bpy ligands. The observed hydrogen bonds can be split into three groups according to the unit type involved in the interaction (Table S4). Adjacent chains form strong interactions along the b axis due to hydrogen bonds between N1 bpy ligand and O52 and O54 outer oxygen atoms as well as N11 bpy molecules and outer O42 and O62 oxygen atoms (Figure 2b). Apart from that, there are two zipper motifs formed by stacking interactions between solely N1 as well as solely N11 bpy molecules. Along the c axis, each cobalt unit is involved in four hydrogen bonds with two adjacent [Co(bpy)_2_(ox)]^+^ moieties (Figure 2c). They are created between both outer oxygen atoms (O82 and O84) and N21 and N31 bpy molecules. This pattern is completed by a zipper formed by O81 oxalate anions and two stacking interactions between bpy ligands (N21-N21 and N31-N31). Along the a axis there is a robust set of hydrogen bonds between alternately arranged cobalt units and chains (Figure 2d). The cobalt units limit the channel size and stabilize the crystal network connecting chains translated along this direction. The hydrogen bonds are created between all ligands of Co(III) units and N1 bpy ligand as well as O61 and O71 oxalate anions from chains and they are reinforced by π-π interactions formed between strongly inclined N1, N31 bpy ligands. In HOF chemistry, usually strong hydrogen bonds are required to stabilize the open frameworks, whereas weak interactions usually are not able to maintain porosity [46]. Therefore, it is worth noting that in the reported case the relatively weak C-H…O bonds are sufficient for structure stabilization and permanent porosity of this system. We can hypothesize that the channels do not collapse for several reasons. First, these interactions are charged assisted according to the definition given by Lopes Jesus and Redinha [50] (“H-bonds wherein the donor group has a positive charge or the acceptor group a negative one are called charge-assisted (CAHBs), ionic, or low-barrier hydrogen bonds”). In our case, oxygen atoms of the oxalate ligand are negatively charged. Moreover, it is proved that weak and labile C-H…N interactions are capable of forming a very complex porous structure [51]. Secondly, multipoint hydrogen bonds are a key factor for the stabilization of MOFs [21]. The presented interactions (Figure 2b–d) clearly indicate that our compound can complete this requirement. Finally, for HOFs stability, additional interactions reinforcing hydrogen bonds and assuring better rigidity are required [46]. Such interactions are driving forces for the preparation of covalent organic frameworks (COFs). In **NCU-1,** we found multiple π-π interactions forming zippers (Figure 2b,c)

In the channels formed by such a crystal network, there are water molecules dispersed over 15 sites. In the final model, their hydrogen atoms are missing and hence, detailed interaction analysis cannot be performed and must be limited to oxygen atoms acting as acceptors of hydrogen bonds. There is only one such interaction, C21-H21…O93[1 + x, y, z]. Taking into account the partial occupancy found for many of the water positions and the lack of efficient bonding, we can expect that they could be easily removed. However, the performed EDX and single crystal experiments showed that the water molecules could not be easily substituted by other solvents (e.g., acetonitrile and bromobenzene).

### 2.3. Thermal Analysis

Thermal decomposition studied by the combined TGA–DTA techniques proceeds in two steps (Figure S6). Loss of crystallization water molecules starts at room temperature (experimental: 8.68%, calculated: 10.48%), and is accomplished at ca. 120 °C. This behavior indicates that the quantity of solvent in the channels may vary and the initial loss of 1.5 water molecules at RT (resulting in 7 water molecules remaining in the system) matches the experimental value ideally. Subsequently, one exothermic and complex step related to a continuous mass loss (67.49%) of oxalate and bipyridine is observed and completed at ca. 400 °C. Finally, a small mass loss occurs and the mass residue corresponds to a mixture of oxides (CuO and Co_x_Fe_y_O_4_; the experimental value is 23.79%, whereas for 7 crystallization water molecules the calculated value is 22.00%). Their presence was confirmed by the powder XRD method registered for products of thermal decomposition. Similar behavior and discrepancy were found for other porous structures indicating that the amount of water in side channels might vary in the broad range [52].

### 2.4. Powder Experiments

**Figure 3:**
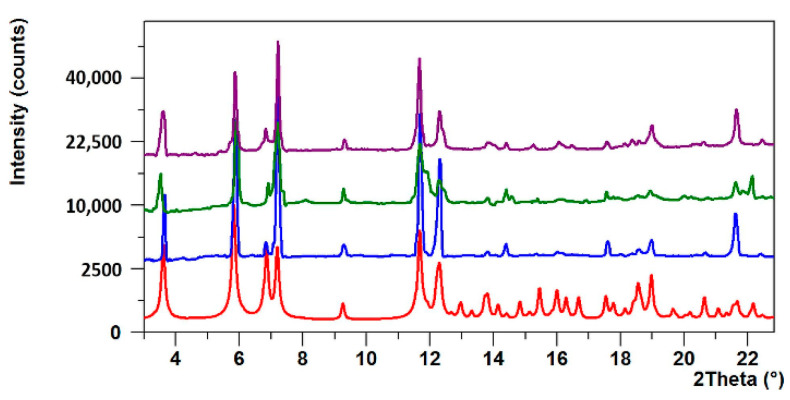
Powder diffractograms for **NCU-1**: calculated pattern (red), pristine sample at room temperature (blue), sample after heating at 120 °C during 3 h (green), and sample after cooling to room temperature (violet). Only the low angle part is presented (3–22.5°).

The PXRD patterns for the dehydrated (3 h in 120 °C) and rehydrated samples (RT) of **NCU-1** show diffraction peaks identical to those observed in the pattern calculated using the single crystal model (Figure 3 and Figure S7). It was shown that careful analysis of certain peak positions could be useful for the determination of the framework stability [52]. The position of the (020) (ca. 3.6° 2θ) and (100) (ca. 5.8° 2θ) reflections can be considered as markers of the channel stability as they are parallel to the c direction. We observed a small shift (ca. 0.1°) of the former peak towards lower angles and to the higher angles (ca. 0.05°) for the latter one. After the heating and cooling cycle, both peaks are present approximately at their starting positions, indicating that this framework is robust and stabilized by charge-assisted hydrogen bonds resulting in the permanent porosity.

### 2.5. Sorption Properties

When water is adsorbed on hydrophilic surfaces, the D’Arcy and Watt (DW) adsorption isotherm can be taken into account [53]. The model assumes that the adsorption of water occurs quite independently, on strong—high-energy and on weak—low-energy sites. The former binding primary centers consist mainly of hydrophilic groups. In the model, it is simplified that there is only one type of secondary sites on which, for the adsorbed water, two-or three-dimensional hydrogen-bonded clusters can begin to build up even before all the primary sites are occupied. The simplest and most popular form of the original DW equation is the sum of Langmuir (adsorption on primary sites) and DS1 isotherms (describing adsorption on secondary sites available for water molecules):

(1) $$a=a_{prim}+a_{\sec}=\underset{L}{\underset{︸}{\frac{a_{mL}K_{L}h}{1/p_{s}+K_{L}h}}}+\underset{DS1}{\underset{︸}{\frac{a_{0}ch}{1-ch}}}\;$$

where *a_mL_* is the total surface concentration of all Langmuir-type (high-energy) sites, *K_L_* is the Langmuir constant, and *h* = *p*/*p_s_*, *a*_0_ and *c* are the number of adsorption centers and the kinetic constant related to the adsorption on secondary sites (of Dubinin–Serpinsky type), respectively.

It is well-known that the difference in the shape of water isotherms is caused by the combined effects of surface hydrophobicity (low-energy centers) and hydrophilicity (high-energy centers). Water is known to have an extremely low affinity toward the “pure” benzene ring and a high affinity toward polar sites leading even to chemisorption. Subsequently, those adsorbed molecules on primary sites (both mentioned above types of centers should be considered) can become secondary ones and adsorption on them occurs following the original Dubinin–Serpinsky mechanism. Thus, the total adsorption is the sum of the adsorption on all available for water molecules, primary (*a_prim_*) and secondary sites (*a*_sec_). This simple model was presented as applicable to the description of different adsorption data (see, e.g., [54,55]).

**Figure 4:**
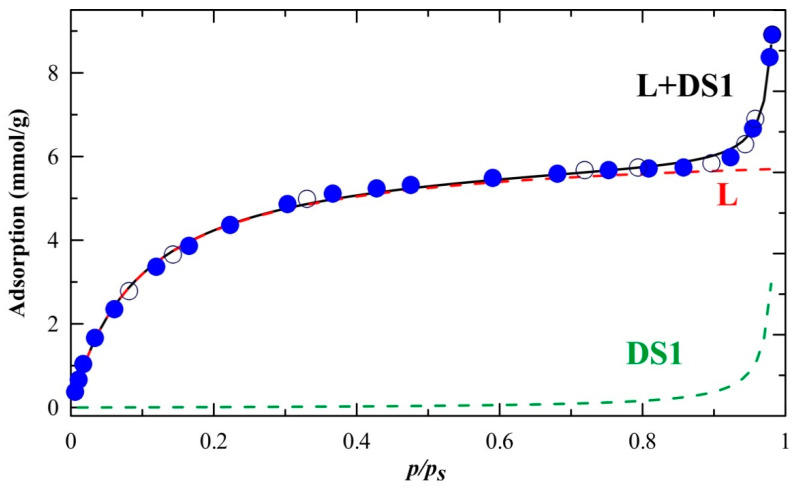
Water adsorption isotherm on **NCU-1**, filled circles—adsorption, empty circles—desorption; solid line—the fit of DW model (Equation (1)), and dashed lines—its components L and DS1.

As **NCU-1** possesses a high amount of high-energy adsorptive centers for the fitting of experimental adsorption data (Figure 4) we have chosen the D-W Equation (1). It is seen that it fits the experimental data satisfactorily—the determination coefficient is 0.9997, the standard error of estimates is low, only in the case of *a*_0_ it exceeds 10% of the estimated value. The fitted values of *K_L_*, c and a_x_ (x = L or 0) are summarized in Table 1. The Langmuir constant *K_L_* is related to the affinity between the adsorbate and the adsorbent, while the parameter c is the ratio of adsorption and desorption constants; value of c = 1 suggests non-interacting sites.

It is seen that practically in the p/p_s_ range 0–0.85 the Langmuir term is sufficient to fit the data properly, the DS1 term is needed only for p/p_s_ > 0.85 (Figure 4). Taking the molar mass of {[Co(bpy)_2_(ox)][{Cu_2_(bpy)_2_(ox)}Fe(ox)_3_]}_n_ without water (it was dehydrated before the adsorption measurement) equal 1306.8 g/mol we obtain a_mL_ equal 8.2 mol H_2_O/mol {[Co(bpy)_2_(ox)][{Cu_2_(bpy)_2_(ox)}Fe(ox)_3_]}_n_) (Table 1) which is very close to the value obtained from the XRD experiments. Thus, one can conclude that H_2_O was adsorbed mainly on primary sites. Moreover, it explained the specific surface area, determined from low temperature adsorption of N_2_, being close to zero.

**Figure 5:**
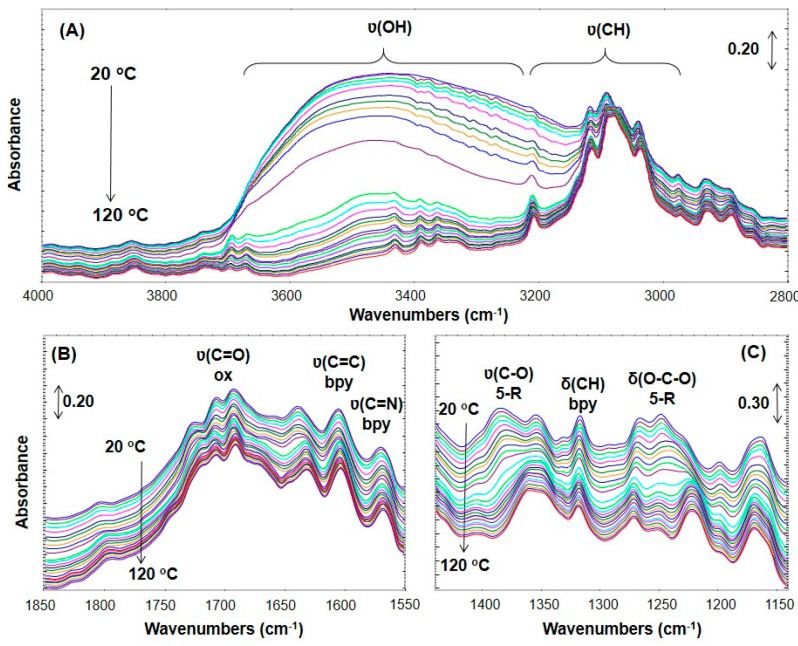
The influence of heat treatment temperature (20–120 °C) in H_2_O/He atmosphere studied via IR spectroscopy. Changes in spectra are shown in 4000–2800 cm^−1^ (**A**), 1850–1550 cm^−1^ (**B**) and 1440–1150 cm^−1^ (**C**) ranges.

To confirm the H_2_O adsorption on primary sites the DRIFT measurements were performed. The IR spectrum of **NCU-1** (Figure 5) shows the characteristic bands of all ligands. Crystallization water molecules can be detected due to the broad intense band with the maximum at 3424 cm^–1^ related to O-H stretching vibrations. The presence of bisbidentately coordinated 2,2′-bipyridine molecules can be confirmed by intense bands at 1600 and 1580 cm^−1^ assigned to ν(C=C) and ν(C=N), respectively, as well as δ(C-H) vibrations in the aromatic ring, whereas medium intensity bands at 3068, 1180, and 1033 cm^−1^ correspond to CH vibrations of aromatic rings [56,57,58,59]. There are four coordination modes of oxalate anions and hence, several bands corresponding to different vibrations of this five-membered ring were registered. Intense bands in the region of 1720–1620 cm^−1^ are related to asymmetric ν_CO_ vibrations, whereas those at 1400–1340 cm^–1^ come from symmetric ν_CO_ vibrations [38,60]. Another bands occurred at 1274, 1246 (ν(CO) + δ(OCO)) cm^−1^.

The increase in temperature causes a gradual removal of H_2_O from the structure—observed mainly as the decrease in the intensity of ν(OH) band (3700–2800 cm^–1^). The band shape being very broad indicates that H-bonding occurs to a great extent. Interestingly, simultaneously to the H_2_O removal from the structure, the ν(CH) bands of bpy ligands become more intense. It proves the strong interactions (through H-bonding) of H_bpy_ with O (from H_2_O). Moreover, these interactions are much stronger than H-bonds between two H_2_O molecules. This observation explains (i) the complete lack of hysteresis loop, (ii) the Langmuir-shape in the low and middle pressure range, and (iii) the drastic rise during pore filling (high pressure range) in H_2_O adsorption isotherm.

Oxalate binds to a metal ion, forming five-membered chelating rings [61,62]. Coordination of oxalate to metal (Me) ions drastically changes its structural and electronic environment, thereby leading to the appearance of more complex IR spectra compared to the free oxalate spectrum [63]. As can be seen from Figure 5C, the position of the IR bands change progressively when H_2_O is removed from the structure. Changes of the five-member ring are more pronounced, while for ν(C=O) are not so obvious. When H_2_O is present in the system, the ring looks to be stressed while it expands (red shift of the IR bands) when removing H_2_O.

**Figure 6:**
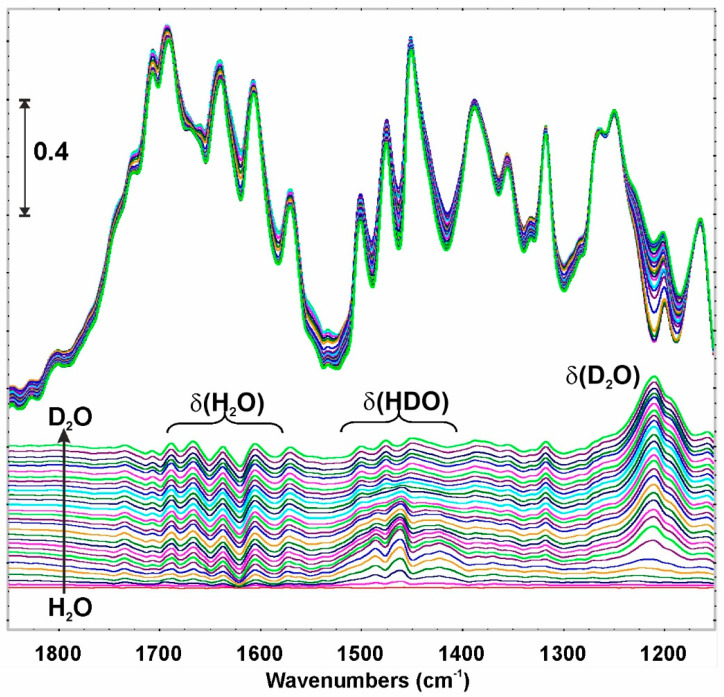
The isotopic effect of H_2_O/D_2_O and D_2_O/H_2_O exchange on the fingerprint region of **NCU-1**. Upper panel the original FTIR spectra, bottom—the differential spectra relative to the spectrum after H_2_O adsorption.

Full reversibility of adsorption H_2_O isotherm (Figure 4) requires a robust structure which cannot undergo a transformation during H_2_O adsorption. Thus H_2_O–D_2_O exchange was studied. The experiments showed that the sorption of both, H_2_O and D_2_O is equal quantitatively (Figure S8). But the most important is that the whole structure remains unchanged—all the characteristic bands mentioned above persist their positions. The only observable spectral changes are connected to H_2_O to D_2_O exchange (Figure 6). Interestingly, the declining δ(H_2_O) band intensities are much smaller than increasing δ(D_2_O). This, together with the facts that: (i) zig-zagged bands appear in δ(H_2_O) region and (ii) some differences in the kinetic of H_2_O and D_2_O desorption from **NCU-1** are observed (Figure S8); is most likely the reflection of the stronger interaction of H_2_O than D_2_O with the structure of **NCU-1**. It is not possible at this moment to distinguish if HDO molecules appear in the channels or as results of proton exchange on the adsorbed molecule.

### 2.6. XAS Experiments

**Figure 7:**
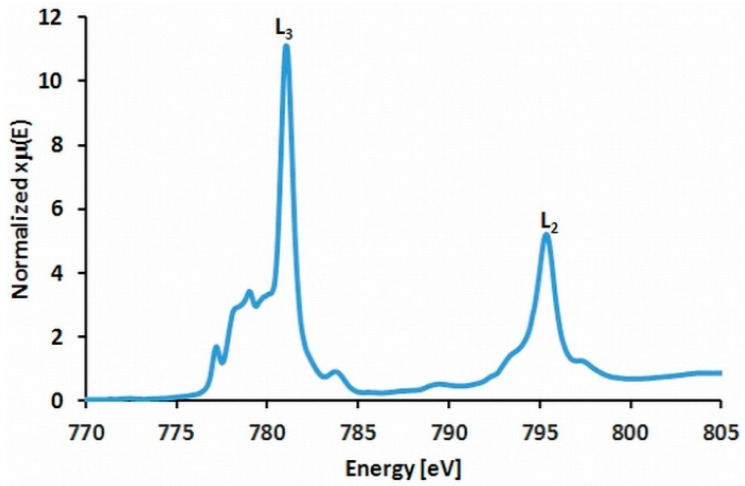
Normalized XANES spectra of cobalt(III) L_2,3_-edge with the multiplet components at 777.2, 779.0 (2p→3d(t_2g_, e_g_)), 781.0 (2p→3d(e_g_)), 783.7 (MLCT), and 795.4 eV.

The energy ranges for iron, cobalt, and copper were carefully selected to avoid the edge regions of other elements, which is crucial for proper spectra normalization. It is especially important for iron which has to be truncated relatively early because of the presence of cobalt. These spectra show a more intense band corresponding to L_3_ edge followed by much smaller L_2_ peaks and the former one is discussed in detail in the Supplementary Materials (Table S5). For iron(III) and copper(II) the observed spectra are similar to those registered for the identical building blocks reported in the literature [64,65] (Figure S9). Hence, we can suppose that the electron structure of those units and the metal oxidation states are also similar (see discussion in Supplementary Materials). For cobalt, the multiplet is complicated and consists of four bands (Table S5). The first two components occurs at 777.2 and 779.0 eV, whereas the main peak is found at 781.0 eV (Figure 7) with a small postedge feature at 783.7 eV. This pattern is similar to [Co(bpy)_3_]^3+^ complex [41]. However, in **NCU-1** one of these ligands is substituted by oxalate anion producing a much weaker ligand field and hence, impairing the direct comparison of multiplets in both complexes. Nevertheless, some observations are useful. In [Co(bpy)_3_]^3+^, the main feature is attributed to the low spin configuration and the weak high energy peak to MLCT transition, whereas two low energy peaks can be ascribed mainly to high spin configuration. Therefore, in the reported compounds it is conceivable that the ligand substitution with oxalate reducing ligand field splitting can result in some admixture of this configuration. However, the magnetic data clearly indicate the diamagnetic low spin state of [Co(bpy)_2_(ox)]^+^ accompanying the active exchange interactions along the [{Cu_2_(bpy)_2_ox}Fe(ox)_3_]^−^ chain counterions.

### 2.7. Magnetic Properties

**Figure 8:**
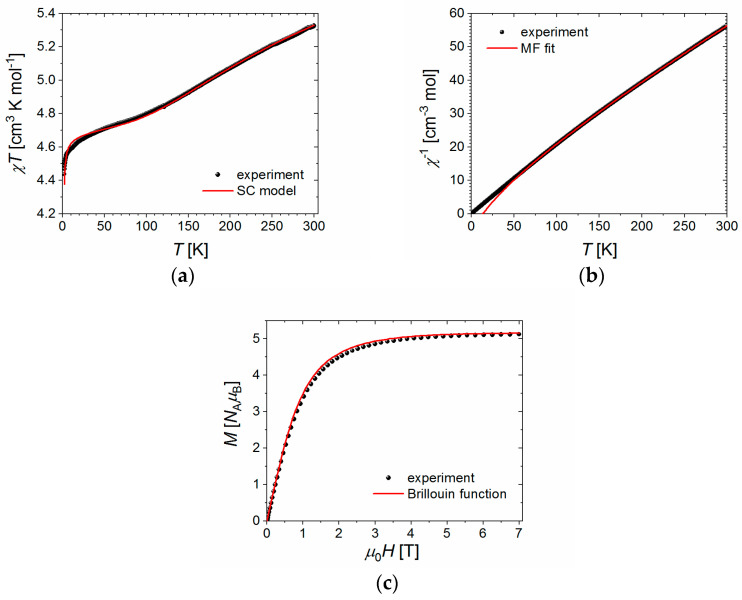
(**a**) Temperature dependence of the χT product of **NCU-1** (symbols) together with the best fit to the semiclassical model (red line). (**b**) Temperature dependence of the inverse molar susceptibility of **NCU-1** (symbols) together with the best fit to the molecular field model (red line). (**c**) Field dependence of the molar magnetization of **NCU-1** at 1.8 K (symbols). The red line shows the Brillouin function for the single Fe(III) spin with S_Fe_ = 5/2 and g_Fe_ = 2.065 (the best fit value implied by the SC model).

Figure 8a shows the plot of the molar magnetic susceptibility in the form of the χT product against temperature T for the powder sample of **NCU-1** (black symbols). The χT values decrease on lowering the temperature from 5.33 cm^3^·K·mol^−1^ at 300 K first steadily and next below 10 K abruptly displaying at the lowest temperatures an upturned kink with the value of 4.44 cm^3^·K·mol^−1^ at 1.98 K and 4.48 cm^3^·K·mol^−1^ at 1.80 K. The high temperature value of χT is slightly higher than the value of 5.13 cm^3^·K·mol^−1^ expected for combined contributions from one Fe(III) ion (S_Fe_ = 5/2, g_Fe_ = 2.0) and two Cu(II) ions (S_Cu_ = 1/2, g_Cu_ = 2.0). Figure 8b shows the inverse susceptibility of the studied system (symbols). On lowering the temperature, it displays an almost linear decrease meeting the origin of the coordinate system (T, χ^−1^). The measurements of the isothermal magnetization demonstrated the lack of opening magnetic hysteresis (not shown) implying a soft magnetic material with negligible anisotropy. In Figure 8c, the experimental data for the field dependence of the magnetization at 1.8 K (symbols) are shown. The curve displays a monotonic increase with an increasing field value, not exceeding the maximal field value level of 5.13 N_A_μ_B_, which points to the strongly antiferromagnetic character of the coupling between the Cu(II) ions leaving effectively the sole contribution from the Fe(III) ion (S_Fe_ = 5/2 with g_Fe_ ≈ 2.065).

In what follows we make an effort to extract the magnetic interactions present in the studied system from the magnetic measurements. Unlike in [4,32], where similar ladder-like topologies have been reported, we attempt, here, to go beyond a simple zero-dimensional (0D) spin Hamiltonian, developed in the next section for the sake of comparison, and reflect the genuine topology of **NCU-1** taking duly into account all interaction pathways (see sections; Molecular field prediction and Semiclassical model).

#### 2.7.1. Simple Dimer Model

The structural analysis of **NCU-1** exhibited the dinuclear “Cu1-ox-Cu2” cations, with an almost symmetrical oxalate bridge forming a ladder due to the connection through tris(oxalato)ferrate(III) ions (Figure 1). To explain the magnetic properties, a simple model involving only copper(II)-copper(II) interaction was first used and copper(II)-iron(III) interactions were ignored. We applied Hamiltonian in the form H = −J_Cu-Cu_(S_Cu1_S_Cu2_). We used the PHI program [66] in the fitting procedure, which allows for the simultaneous fitting of χT(T) and M(H) dependences. In this simple model, temperature-independent magnetism (χ_0_) and molecular field corrections term (zJ’) were also introduced. Two different approaches to g-factors were used. In the first, only one g-factor was used. The best reasonable fit parameters were g_Cu_ = g_Fe_ = 2.068, J_Cu-Cu_ = −324.4 cm^−1^, χ_0_ = +0.00089 cm^3^·mol^−1^, zJ’ = +0.007 cm^−1^ and with good values of the test functions R_χT_ = Σ (χT_obs_ − χT_calc_)^2^/Σ (χT_obs_)^2^ = 3.66·10^−5^ for χT(T) data and R_M_ = Σ (M_obs_ − M_calc_)^2^/Σ (M_obs_)^2^ = 4.54·10^−4^ for M(H) data. In the second, g-factors, g_Cu_ = 2.00 and g_Fe_ = 2.065, were taken from SC model (see below) and fixed. In this case, the best reasonable fit parameters were J_Cu-Cu_ = −327.0 cm^−1^, χ_0_ = +0.00095 cm^3^·mol^−1^, zJ’ = –0.006 cm^−1^ and with following values of the test functions R defined as above, R_χT_ = 3.17·10^−5^ for χT(T) data and R_M_ = 4.31·10^−4^ for M(H) data. As we can see, in both cases, the key parameter, i.e., the Cu-Cu exchange integral, has shown almost identical values.

#### 2.7.2. Molecular Field (MF) Prediction

**Figure 9:**
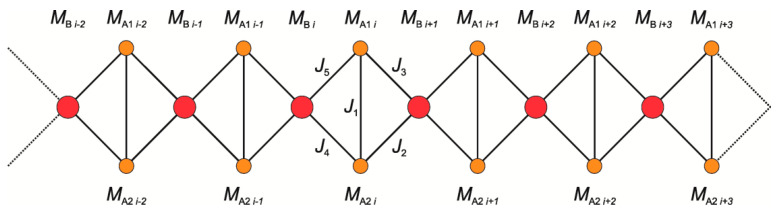
Scheme of a chain with A_1_A_2_B topology. The system consists of alternating single-center subsystems of type B and two single-center subsystems of type A. The molecular field theory is expressed in terms of net magnetization $M_{A_{1}i}$, $M_{A_{2}i}$ and $M_{Bi}$ (*i* = 1, …, N).

Let us consider a chain with A_1_A_2_B topology, as depicted in Figure 9, where single centers of spin S_B_ with the spectroscopic factor g_B_ alternate with a couple of spins S_A_ with the spectroscopic factor g_A_. Centers A_1_, A_2_ and B are exchange coupled with J_1_, J_2_, J_3_, J_4_, and J_5_ being the exchange coupling constants. The chain is of length N in terms of the A_1_A_2_B units. The pertinent Hamiltonian has the following form

(2) $$\begin{array}{cc} \hat{H}=\sum_{i=1}^{N} & \{-J_{1}{\hat{S}}_{A_{1}i}\cdot {\hat{S}}_{A_{2}i}-J_{2}{\hat{S}}_{A_{2}i}\cdot {\hat{S}}_{Bi+1}-J_{3}{\hat{S}}_{A_{1}i}\cdot {\hat{S}}_{Bi+1}-J_{4}{\hat{S}}_{A_{2}i}\cdot {\hat{S}}_{Bi}-J_{5}{\hat{S}}_{A_{1}i}\cdot {\hat{S}}_{Bi}+\\ & g_{A}\mu_{B}({\hat{S}}_{A_{1}i}+{\hat{S}}_{A_{2}i})\cdot \vec{H}+g_{B}\mu_{B}{\hat{S}}_{Bi}\cdot \vec{H}\} \end{array}$$

where $\mu_{B}$ is the Bohr magneton, $\vec{H}$ denotes the external magnetic field, and the periodic boundary conditions are imposed, i.e., ${\hat{S}}_{BN+1}\equiv {\hat{S}}_{B1}$. The molecular field theory is expressed in terms of net magnetization $M_{A_{1}i}=-N_{A}g_{A}\mu_{B}\langle {\vec{S}}_{A_{1}i}\rangle$, $M_{A_{2}i}=-N_{A}g_{A}\mu_{B}\langle {\vec{S}}_{A_{2}i}\rangle$ and $M_{Bi}=-N_{A}g_{B}\mu_{B}\langle {\vec{S}}_{Bi}\rangle$ (molar quantities are considered here and angle brackets denote thermodynamical averaging) corresponding to the i-th subsystem of A-type and B-type, respectively, rather than the subsequent spin variables.

Applying the procedure described in Supplementary Information, and identifying sublattices A_1_ and A_2_ with the Cu(II) subsystem (S_Cu_ = 1/2) and sublattice B with the Fe(III) subsystem (S_Fe_ = 5/2), one arrives at the following practical formula for the MF molar susceptibility of the studied system χ_MF_ = P/Q, where

(3a) $$\begin{array}{cc} P & =\frac{N_{A}\mu_{B}^{2}}{k_{B}T}\{\frac{1}{2}g_{\mathrm{Cu}}^{2}+\frac{35}{12}g_{\mathrm{Fe}}^{2}+\frac{35}{24}g_{\mathrm{Cu}}g_{\mathrm{Fe}}\frac{J_{24}+J_{35}}{k_{B}T}+\frac{1}{8}g_{\mathrm{Cu}}^{2}\frac{J_{1}}{k_{B}T}+\\ & +\frac{35}{192}\frac{2g_{\mathrm{Cu}}g_{\mathrm{Fe}}J_{1}(J_{24}+J_{35})-g_{\mathrm{Fe}}^{2}J_{1}^{2}-g_{\mathrm{Cu}}^{2}{\left(J_{24}-J_{35}\right)}^{2}}{{\left(k_{B}T\right)}^{2}}\} \end{array}$$

(3b) $$Q=1-\frac{1}{96}\left[\frac{70(J_{24}^{2}+J_{35}^{2})+6J_{1}^{2}}{{\left(k_{B}T\right)}^{2}}+\frac{35J_{1}J_{24}J_{35}}{{\left(k_{B}T\right)}^{3}}\right]$$

where k_B_—the Boltzmann constant, J_24_ = J_2_ + J_4_, and J_35_ = J_3_ + J_5_. The above formula will be used in what follows to obtain a preliminary estimate of the exchange interactions in the system under study.

#### 2.7.3. Semiclassical (SC) Model

There is no available exact model to treat such a complex system with a knotted arrangement of spins within the chain. Nevertheless, even a necessarily approximate approach aimed at rationalizing the magnetic behavior may shed some light on the nature of magnetic interactions in this system. To obtain the zero-field susceptibility of the chain segment, a semiclassical analytical approach [67] is employed. In this scheme, the relatively large spin of the Fe(III) ion of the FeCu_2_ unit (S_Fe_ = 5/2) is treated as a classical commuting variable, while the remaining two Cu(II) spins (S_Cu_ = 1/2) are given a rigorous quantum mechanical treatment. Unfortunately, the ensuing calculation cannot be performed analytically for the configuration of exchange couplings depicted in Figure 9, representing one of the pathological situations mentioned by the authors of [67]. Only by accepting the further restriction of J_2_ = J_3_ ≡ J_23_ and J_4_ = J_5_ ≡ J_45_ the calculation can be brought to its final explicit conclusion. The Hamiltonian of the finite chain involving N trimer units in Equation (2) may be rewritten as the sum of partial Hamiltonians

(4) $${\hat{H}}_{N}=\sum_{i=1}^{N}{\hat{H}}_{i,i+1}(\Psi_{i},{\vec{S}}_{i},{\vec{S}}_{i+1},\vec{H})$$

where $\Psi_{i}$ denotes the quantum subsystem of the two Cu(II) ions within the i-th trimer, ${\vec{S}}_{i}$ denotes the classical spin of the Fe(III) ion of that trimer, and $\vec{H}$ is the external magnetic field, and the partial Hamiltonians real

(5) $${\hat{H}}_{i,j+1}=({\hat{S}}_{Cu_{1}i}+{\hat{S}}_{Cu_{2}i})(-J_{45}{\vec{S}}_{i}-J_{23}{\vec{S}}_{i+1}+g_{Cu}\mu_{B}\vec{H})-J_{1}{\hat{S}}_{Cu_{1}i}\cdot {\hat{S}}_{Cu_{2}i}+g_{Fe}\mu_{B}{\hat{S}}_{i}\cdot \vec{H}$$

where isotropic Heisenberg exchange interactions between the constituent ions are assumed and the Zeeman terms introduced. In addition to the coupling constant J_1_, describing the superexchange interaction between the two Cu(II) ions of the trimer unit, two different superexchange coupling constants J_23_(= J_2_ = J_3_) and J_45_(= J_4_ = J_5_) alternating along the chain are assumed. The corresponding partition function may be written as

(6) $$Z_{N}(\vec{H})=\int d{\vec{S}}_{1}\int d{\vec{S}}_{2}\ldots \int d{\vec{S}}_{N+1}V_{1,2}({\vec{S}}_{1},{\vec{S}}_{2},\vec{H})\ldots V_{N,N+1}(\vec{S},{\vec{S}}_{N+1},\vec{H})$$

where $V_{i,i+1}({\vec{S}}_{i},{\vec{S}}_{i+1},\vec{H})=Tr\{\exp[-\beta {\overset{⌢}{H}}_{i,i+1}(\Psi_{i},{\vec{S}}_{i},{\vec{S}}_{i+1},\vec{H}]\}$ and the trace is performed over the degrees of freedom of the quantum subsystem $\Psi_{i}$. In Equation (6) $\int d{\vec{S}}_{i}$ denotes integrating over all directions available to the classical variable ${\vec{S}}_{i}$ and $\beta =1/k_{B}T$ is the Boltzmann factor. The zero-field magnetic susceptibility is obtained through second-order differentiation of $Z_{N}(\vec{H})$ with respect to $\vec{H}$. As argued in [67], if only the exchange couplings in the system are isotropic, the calculation can be performed rigorously. Taking in the final step the thermodynamic limit (N→∞) one obtains the following expression for the zero-field susceptibility of the FeCu_2_ chain unit

(7) $$\begin{array}{ll} \chi_{\mathrm{SC}}=\frac{N_{A}\mu_{B}^{2}}{3k_{B}T}\{ & g_{\mathrm{Fe}}^{2}S_{\mathrm{Fe}}(S_{\mathrm{Fe}}+1)\frac{1+\rho }{1-\rho }+6g_{\mathrm{Cu}}^{2}{\rho^{\prime }}_{0}+\\ & +2g_{\mathrm{Cu}}^{2}S_{\mathrm{Fe}}(S_{\mathrm{Fe}}+1)\beta^{2}[(J_{23}^{2}+J_{45}^{2}){\rho^{\prime \prime }}_{0}+2J_{23}J_{45}{\rho^{\prime \prime }}_{1}]+\\ & +4g_{\mathrm{Fe}}g_{\mathrm{Cu}}S_{\mathrm{Fe}}(S_{\mathrm{Fe}}+1)\frac{1}{1-\rho }\beta (J_{23}+J_{45})({\rho^{\prime }}_{0}+{\rho^{\prime }}_{1})+\\ & +8g_{\mathrm{Cu}}^{2}S_{\mathrm{Fe}}(S_{\mathrm{Fe}}+1)\frac{1}{1-\rho }\beta^{2}(J_{23}{\rho^{\prime }}_{0}+J_{45}{\rho^{\prime }}_{1})(J_{23}{\rho^{\prime }}_{1}+J_{45}{\rho^{\prime }}_{0})\} \end{array}$$

where the quantities *ρ*, *ρ_α_*’, and *ρ_α_*” (α=0,1) are dimensionless functions of J_1_, J_23_, J_45_, and *β*, and their definitions are provided in the Supporting Information. There is no rigorous way one can introduce the interchain interactions into the model. Therefore, we decide here to employ the molecular field approximation, which yields the molar susceptibility of the interacting FeCu_2_ chain unit $\chi_{CCU}$ (CCU stands for Coupled Chain Unit) in the following form

(8) $$\chi_{\mathrm{CCU}}=\frac{\chi_{\mathrm{SC}}}{1-\frac{zJ^{\prime }\chi_{\mathrm{SC}}}{N_{A}\mu_{B}^{2}{\bar{g}}^{2}}}$$

where zJ′ is the effective interchain coupling constant, and $\bar{g}=\sqrt[{3}]{g_{Cu}^{2}g_{Fe}}$ is the average spectroscopic factor of the FeCu_2_ chain unit.

#### 2.7.4. MS and SC Results

Let us start the presentation of the results with the analysis of the high-temperature behavior of the χ^−1^ signal by employing the molecular field theory. The MF theory was demonstrated above to give rise to the specific prediction for the high-temperature regime quoted in Equation (3a,b). The function ${\left(\chi_{MF}+\chi_{0}\right)}^{-1}$ was fitted to the experimental data of the inverse susceptibility above 80 K. All parameters g_Cu_, g_Fe_, J_1_, J_24_, J_35_, and χ_0_ were relaxed during the fitting run. The best fit shown in Figure 8b by the solid line yielded the agreement quotient of

(9) $$R_{\chi^{-1}}=\sum_{T>80K}{\left[\chi_{\exp}^{-1}(T)-\chi_{\mathrm{mod}el}^{-1}(T)\right]}^{2}/\sum_{T>80K}{\left[\chi_{\exp}^{-1}(T)\right]}^{2}=1.5\cdot 10^{-7}$$

and the following set of parameters: g_Cu_ = 1.9(1), g_Fe_ = 2.063(2), J_1_ = −226(51) cm^−1^, J_24_ = −20(1) cm^−1^, J_35_ = −20(2) cm^−1^, and χ_0_ = +0.0014(1) cm^3^·mol^−1^. The Landé factors are plausible for the Cu(II) and Fe(III) ions, although that of the Cu(II) ions is slightly reduced. There is a large antiferromagnetic superexchange coupling between the Cu(II) ions, and relatively smaller and, also, antiferromagnetic couplings between the Cu(II) and Fe(III) ions on the order of −10 cm^−1^ (let us remind you that J_24_ = J_2_ + J_4_ and J_35_ = J_3_ + J_5_). Let us note that the amplitudes of the J_24_ and J_35_ are exactly the same not allowing to differentiate between the bridges joining the Cu1 and Cu2 ions with the Fe ion. The temperature-independent susceptibility correction χ_0_ turns out to be positive with the amplitude comparable to that reported for the Co(III) ion in SrTi_0.65_Co_0.35_O_3_ (~0.00081 cm^3^·mol^−1^) by Pascanut et al. [68] and in oligonuclear Fe(III)-Co(III) compound with oxido-, sulfato-, and cyanido-bridging ligands (~0.000985 cm^3^·mol^−1^) [69].

Preliminary calculations of the χT signal using the SC chain model with an additive temperature-independent susceptibility correction χ_0_ showed a large discrepancy between the experimental and calculated values (the calculated signal was substantially larger in the low temperature regime). This indicated the need to take into account the interchain interactions. They originate from the system of hydrogen bonding, π-π stacking contacts, and finally from the through-space dipole-dipole coupling of the constituent magnetic moments. The reality-faithful introduction of the interchain coupling into the procedure would pose a cumbersome and complex computational problem, therefore we decided to conduct it within the simple and well-known molecular field approximation, where the resultant susceptibility signal is calculated according to the formula in Equation (8). The trials to relax the whole set of parameters, g_Cu_, g_Fe_, J_1_, J_23_(= J_2_ = J_3_), J_45_(= J_4_= J_5_), χ_0_, and zJ’ were all unsuccessful failing to reproduce the low-temperature end of the χT signal, although the values of the coupling constants J_23_ and J_45_ fell very close. Therefore, we decided to reduce the number of fitted parameters by setting these coupling constants equal. Just thereon we observed an instant improvement of the fit quality resulting in an excellent agreement of the model with the experimental data, however it involved an unphysical prediction of g_Cu_ = 1.28(4), and, therefore, must be dismissed. In the next and final step, we carried out the fitting procedure with the Landé factor of the Cu(II) ions fixed at the value of g_Cu_ = 2.0. The ensuing fit is shown in Figure 8a by the solid line and yielded the satisfactory agreement quotient of

(10) $$R_{\chi T}=\sum_{i}{\left[{\left(\chi T\right)}_{i}^{\exp}-{\left(\chi T\right)}_{i}^{\mathrm{mod}el}\right]}^{2}/\sum_{j}{\left[{\left(\chi T\right)}_{j}^{\exp}\right]}^{2}=4.3\cdot 10^{-6}$$

and the following parameter values: g_Fe_ = 2.0650(3), J_1_ = −275(29) cm^−1^, J_23_ = J_45_ = −3.8(1.6) cm^−1^, χ_0_ = 0.00105(5) cm^3^·mol^−1^, zJ’ = −0.0273(5) cm^−1^. The value of the Landé factor of the Fe(III) ion is consistent with molecular field prediction. The exchange coupling constant between the Cu(II) ions J_1_ is negative and larger in magnitude than its MF counterpart, but their values agree within the error margins (their error intervals do overlap). The values of the coupling constants J_23_ = J_45_ between the Cu(II) and Fe(III) ions are smaller than the MF prediction of −10 cm^−1^, however the MF model usually tends to overestimate the coupling strength. The susceptibility correction χ_0_ is likewise positive and smaller, which makes it closer to the values reported in the literature [68,69]. And finally, the interchain coupling constant is of plausible magnitude and negative, which implies an antiferromagnetic arrangement of magnetic moments originating in adjacent chains. The solid line in Figure 8c shows the shape of the Brillouin function corresponding to a single Fe(III) ion (S_Fe_ = 5/2) with the Landé factor equal to the best fit value g_Fe_ = 2.065. A good agreement is consistent with the strong antiferromagnetic coupling between the Cu(II) ions. A small discrepancy is observed at intermediate field values, most probably due to the state of weak frustration, which is the case with all coupling constants J_1_ to J_5_ being negative.

#### 2.7.5. Magnetostructural Comparison

In the reported structure, copper(II) cations are close to each other (5.132 Å) and the bridge geometry given by coplanar arrangement of Cu1 and Cu2 basal planes (12.57°) as well as angles between basal planes and bridging oxalate (3.40 and 9.54°) result in strong antiferromagnetic Cu-Cu coupling (−275 cm^−1^). Table S2 shows a list of compounds containing the oxalate-bridged Cu(II) dimer units together with the corresponding values of the Cu(II)-Cu(II) exchange coupling constant. It can be seen that the magnetic coupling ranges from −400 cm^−1^ to weak antiferromagnetic or ferromagnetic coupling. The studied compound falls onto the weaker side of the strong antiferromagnetic coupling, being most close to compounds **3** or **38**. It is well established for oxalate-bridged Cu(II) complexes [70,71,72] that the value and type of the exchange coupling is essentially determined by the magnitude of the overlap between the symmetry-adapted highest occupied molecular orbitals of the oxalate ligand and the metal-centered magnetic orbitals. If the four oxalate oxygen atoms are coordinated with short bond distances to each Cu(II) center in a way that is coplanar with the singly occupied molecular orbitals (SOMOs) of the copper atoms (coplanar topology) the strong antiferromagnetic coupling arises. On the other hand, if the oxalate ligand behaves as an asymmetric bis-bidentate bridging ligand and the two metal-centered magnetic orbitals are parallel to each other and perpendicular to the oxalate ligand (perpendicular topology), a weak ferromagnetic (accidental orthogonality) or antiferromagnetic coupling is implied. The full discussion of the geometric parameters affecting the bridge geometry, and hence, possibly influencing magnetic properties is given in the Supplementary Materials (Figures S10–S14, Table S2).

## 3. Materials and Methods

### 3.1. Materials and General Procedure

The starting K_3_[Fe(ox)_3_]·3H_2_O [73] and [Cu(bpy)_2_(NCS)](NO_3_) [74] complexes were prepared according to the literature procedures. Other reagents used in the synthesis were of analytical grade and used without further purification. Elemental analysis (C, H, N) was carried out with a Vario MACRO analyser. IR spectrum was recorded on a Perkin Elmer FT-IR 2000 spectrophotometer in the 4000–400 cm^−1^ region using KBr discs. Thermal behavior was studied on an SDT 2600 TA Instruments by simultaneous thermogravimetric analysis (TGA) and differential thermal analysis (DTA) in a stream of synthetic air up to 1000 °C. The powder diffraction patterns were collected using a Phillips X’Pert Pro diffractometer equipped with an X’Celerator Scientific RTMS detector using CuK_α_ radiation. The diffractograms were registered with step of 0.017° 2θ and exposure time 300 s in the range 3 < 2θ < 50° for **NCU-1** and 180 s in the range 5 < 2θ < 120° for the products of the thermal analysis. X-ray absorption spectra were recorded at the National Synchrotron Radiation Centre SOLARIS at the bending magnet PEEM/XAS beamline for Fe (680–770 eV), Co (750–920 eV) and Cu (900–1050 eV) L_2,3_-edges. The sample was finely ground and attached to double-sided adhesive conductive graphite tape. The measurements were performed with the step size of 0.2 eV for the pre-edge region, 0.1 eV for the edge regions, and 0.5 eV for the high energy part. The data sets were collected at room temperature in ultra-high vacuum (UHV) using total electron yield mode (TEY). The measurements were repeated at least five-fold. The data were processed using the ATHENA program from Demeter package [75]. Magnetic measurements in the temperature range 1.8–300 K (µ_0_H = 0.1 T) and in the magnetic field range 0–7 T (T = 1.8 K) were performed using a SQUID MPMS-3 magnetometer, in a sample holder made from a small plastic envelope with very well estimated diamagnetic characteristics. The raw data have been corrected for the diamagnetic contribution of the sample holder measured in a separate run. The diamagnetic susceptibility of the studied compound estimated using Pascal constants was found to amount to −660 × 10^−6^ cm^3^·mol^−1^, however, it was not subtracted from the measured susceptibility. Instead, it will be taken into account within the following fitting procedures in the form of an additive correction χ_0_ together with the expected positive temperature-independent contribution (TIP, the second-order Zeeman contribution) from the off-chain Co(III) diamagnetic ion and two Cu(II) ions.

### 3.2. Synthesis of {[Co(bpy)_2_(ox)][{Cu_2_(bpy)_2_ox}Fe(ox)_3_]}_n_·8.5nH_2_O 1

First, 0.0971 g (ca. 0.41 mmol) CoCl_2_∙6H_2_O and 0.1684 g (ca. 0.43 mmol) K_3_[Fe(ox)_3_]∙3H_2_O were placed in a beaker and dissolved in 10 mL of distilled water yielding orange solution which was added dropwise to a green solution of 0.1970 g (ca. 0.40 mmol) [Cu(bpy)_2_NCS](NO_3_) dissolved in 45 mL of acetonitrile. After 2–3 weeks, salmon needles of **NCU-1** appeared. They were isolated by filtration and identified by SC XRD analysis. The number Yield: 73.0 mg. Found: C, 41.59; H, 3.17; N, 7.73%. Calc. for C_50_H_49_CoCu_2_FeN_8_O_28.5_: C, 41.14; H, 3.38; N, 7.68%. IR/cm^–1^: 3424 (br, s), 3116 (w), 3068(m), 3037 (w), 2921 (m), 1725 (s), 1657 (vs), 1620 (vs), 1605 (w), 1571 (m), 1499 (m), 1474 (s), 1444 (s), 1384 (m), 1345 (s), 1317 (s), 1274 (m), 1246 (w), 1224 (m), 1164 (s), 1104 (s), 1033 (s), 981 (s), 881 (vs), 855 (m), 771 (vs), 728 (m), 656 (s), 618 (s), 529 (s), 476 (vs), 421 (m). After another month, the green crystals were grown from the supernatant and identified by SC XRD as [Cu(bpy)(NCS)_2_] (see the discussion below).

### 3.3. Single Crystal X-ray Diffraction Measurements

The preliminary experiments were carried out at room temperature using an Oxford Diffraction Sapphire CCD diffractometer, MoKα radiation λ = 0.71073 Å. However, due to the large b value, a significant spot overlapping was observed, and hence, the symmetry and this cell parameter were erroneously identified as P2/c and 24.682(4) Å, respectively. The final diffraction data of the studied compound were collected at 100 K on MX14-2 beamline (Helmholtz Zentrum Berlin, Bessy II). First, the data were processed in xdsapp software [76,77], and subsequently CrysAlis Pro [78] was used to apply the numerical absorption correction. The structure was solved by the direct methods and refined with the full-matrix least-squares procedure on F^2^ (SHELX 2013/4) [79]. All heavy atoms were refined with anisotropic displacement parameters. Positions of hydrogen atoms were assigned at the calculated positions with thermal displacement parameters fixed to a value of 20% higher than those of the corresponding carbon atoms. There are 15 water positions accounting for 8.5 water molecules given in the formula. ISOR restraints were applied for some atoms, mainly partially occupied water molecules to assure stable refinement. All figures were prepared in DIAMOND and ORTEP-3 [80,81]. The results of the data collection and refinement have been summarized in Table S1. Additional single crystal experiments were performed also at MX14-2 beamline for crystals soaked over 48–72 h in acetonitrile and bromobenzene.

### 3.4. Water Adsorption-Desorption Measurements

The detailed specification of the implemented procedure, was described elsewhere [82,83,84]. Briefly, water adsorption-desorption measurements were performed at the temperature of 25 °C using a typical gravimetric adsorption apparatus equipped with the Baratron pressure transducers (MKS Instruments, München, Germany). Before the measurements, each sample was thermally desorbed under a high vacuum until a constant mass was obtained (usually after 3 days).

### 3.5. In-Situ DRIFT Investigations

Water adsorption was performed under isobaric conditions (*p* = 4 kPa by flowing He gas through H_2_O scrubber at 25 °C). By changing the temperature of the chamber (A Praying Mantis in-situ cell from Harrick Scientific Corporation) up to 120 °C (with the ramp 1 °C/min), the adsorption isobars were obtained. The construction of this cell enables the thermal treatment of the powdered sample up to 600 °C in any controlled atmosphere or in vacuum. The IR spectra of the samples were recorded (using Nicollet S10, Thermo Scientific, Waltham, MA, USA) with a period of 2 min.

## 4. Conclusions

Tending to develop new multimetallic coordination complexes [85,86], we report the original architecture {[Co(bpy)_2_(ox)][{Cu_2_(bpy)_2_(ox)}Fe(ox)_3_]}_n_·8.5nH_2_O **NCU-1**, as a product of the self-assembly between the complex building block originating from the recombination of [Co(H_2_O)_6_]Cl_2_, [Cu(bpy)_2_NCS](NO_3_) and K_3_[Fe(ox)_3_] in aqueous solution. The molecular recognition led to the occurrence of oxalate-bridged 1D [{Cu_2_(bpy)_2_(ox)}Fe(ox)_3_]^−^_∞_ ladders of very rare topology, shown only very recently to crystallize with simple alkali metal cations. In **NCU-1**, however, these molecular 1D modules co-crystallize with much larger in situ formed [Co(bpy)_2_(ox)]^+^ cations to produce a new robust porous architecture stabilized by charge-assisted hydrogen bonds and π-π interactions. The sorption studies coupled with the IR studies indicated the full reversibility of quantitative sorption/desorption of H_2_O over the hydrophilic surface within the porous space 963.7 Å^3^ per one cell (16.9% of cell volume). Thus, **NCU-1** exhibits an interesting hybrid porous molecular system combining the structural features of MOFs and HOFs. Finally, considering a relatively complicated 1D ladder topology of **NCU-1**, we developed our own general approach to optimize the description of super-exchange magnetic interactions in the like systems. The presented studies could be important from the viewpoint of a still developing field of multicomponent modular systems.

**Figure 10:**
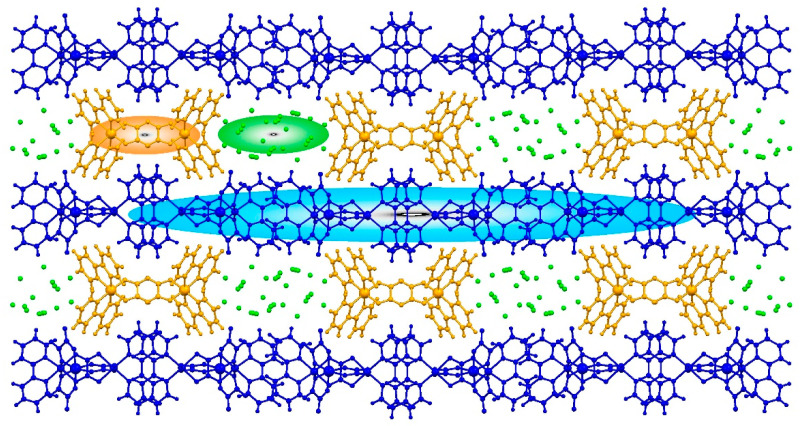
The partition of the molecular architecture of **NCU-1** for the modular structure-function modification. Yellow zones provide the space for various functional cations, the green zones provide the solvent accessible space, and the blue zones provide the modification of the extended coordination skeleton. For more details see text.

The presented studies could be important from the viewpoint of the still developing field of multicomponent modular systems. While the individual molecular architecture of **NCU-1** seemed to be unavailable using the direct self-assembly method from the building blocks, we conclude that its overall scheme may be advantageous towards the creative design through the systematic modular structure-property modification approach. Each of the **NCU-1** components, namely anionic bimetallic chain, monometallic cationic complex, and crystallization solvent molecules creates its own specific zone (Figure 10). It is quite easy to imagine that other cationic species of the functional potential, e.g., the spin-crossover complexes (switchability) or non-innocent organic cations (electronic conductance) may be arranged in the dedicated 1-dimensional space (orange zone in Figure 10) to shape the separate functional subnetwork. The implementation of the building blocks of diverse size or/and electronic charge should implicate, on the other hand, the tuning of solvent accessible space zone, to reinforce the sorption potential (green zone in Figure 10). This functionality might be also built up by a systematic use of accessible oxalato ligands analogues, e.g., broad family of anilato ligands, through the formation of analogous (or modified) coordination arrangement (blue zone in Figure 10).

## Figures and Tables

**Table 1:** The estimates of model parameters for water adsorption on **1**.

Parameter	Estimate	Std. Error	*p*-Value
*a*_mL_ [mmol/g]	6.259	0.074	7.7 × 10^−25^
*K*_L_	3.27	0.15	2.5 × 10^−14^
*C*	1.0073	0.0016	1.8 × 10^−40^
*a*_0_ [mmol/g]	0.0386	0.0052	7.6 × 10^−7^
*R* ^2^	0.9997		

## Data Availability

Data is contained within the article or supplementary material.

## Supplementary Materials

The supporting information can be downloaded at: https://www.mdpi.com/article/10.3390/ijms23031556/s1.
