# Supplementary material for: Sorption and Magnetic Properties of Oxalato-Based Trimetallic Open Framework Stabilized by Charge-Assisted Hydrogen Bonds
Source: Int J Mol Sci. 2022 Jan 29;23(3):1556. doi: 10.3390/ijms23031556 (PMC8835875; doi:10.3390/ijms23031556)

checkCIF/PLATON report

Structure factors have been supplied for datablock(s) be2-auto-sphere

THIS REPORT IS FOR GUIDANCE ONLY. IF USED AS PART OF A REVIEW PROCEDURE FOR PUBLICATION, IT SHOULD NOT REPLACE THE EXPERTISE OF AN EXPERIENCED CRYSTALLOGRAPHIC REFEREE.

No syntax errors found. CIF dictionary Interpreting this report

Datablock: be2-auto-sphere

Bond precision:	C-C = 0.0050 A	Wavelength=0.79990	
Cell:	a=15.4103(3)	b=49.1416(9)	c=7.6343(2)
	alpha=90	beta=100.402(2)	gamma=90
Temperature:	100 K		

	Calculated	Reported
Volume	5686.3(2)	5686.4(2)
Space group	P 21/c	P 21/c
Hall group	-P 2ybc	-P 2ybc
Moiety formula	2(C28 H16 Cu2 Fe N4 O16), 2(C22 H16 Co N4 O4), 15.1(O0.25), 4.4	(Co Cu2 Fe 10+), (N2 C10 H8)4, (C2 O4 2-)5, (H2 O)8.5
Sum formula	C100 H64 Co2 Cu4 Fe2 N16 O57	C50 H49 Co Cu2 Fe N8 O28.50
Mr	2885.43	1459.83
Dx,g cm-3	1.685	1.705
Z	2	4
Mu (mm-1)	1.889	1.823
F000	2908.0	2976.0
F000'	2915.19	
h,k,lmax	19,61,9	19,61,5
Nref	11641	8676
Tmin,Tmax	0.858,0.913	0.712,0.914
Tmin'	0.694	

Correction method= # Reported T Limits: Tmin=0.712 Tmax=0.914
AbsCorr = SPHERE

Data completeness= 0.745 Theta(max)= 29.996

R(reflections)= 0.0406(7384) wR2(reflections)= 0.1216(8676)

S = 1.056 Npar= 876

The following ALERTS were generated. Each ALERT has the format
test-name_ALERT_alert-type_alert-level.
Click on the hyperlinks for more details of the test.

Alert level A

PLAT029_ALERT_3_A _diffrn_measured_fraction_theta_full value Low . 0.774 Why?

Author Response: This is false alert, because in the report (given below) from CrysAlis the completeness of the data set is 97.3% for resolution up yo 0.83 A resolu- # # # % average mean mean tion(A) kept theory unique compl redundancy F2 F2/sig(F2) Rint RsigmaB -- inf-1.50 13342 1809 1795 99.2 7.4 21920.16 29.20 0.036 0.019 1.50-1.19 13341 1801 1795 99.7 7.4 10573.98 15.97 0.056 0.036 1.19-1.04 11837 1831 1795 98.0 6.6 6126.69 10.31 0.081 0.059 1.04-0.94 12567 1861 1795 96.5 7.0 5146.85 9.07 0.102 0.068 0.94-0.87 12501 1860 1795 96.5 7.0 4272.32 7.80 0.117 0.080 0.87-0.82 12342 1888 1795 95.1 6.9 3106.30 6.17 0.150 0.103 0.82-0.78 12254 1873 1795 95.8 6.8 2404.37 4.94 0.196 0.125 0.78-0.74 12085 1934 1795 92.8 6.7 1557.31 3.69 0.324 0.178 0.74-0.71 11781 1918 1795 93.6 6.6 1055.10 2.70 0.469 0.260 0.71-0.66 7508 3764 1802 47.9 4.2 580.48 1.22 0.705 0.545 -- inf-0.66 119558 20539 17957 87.4 6.7 6085.30 9.69 0.091 0.059 inf-0.80 80917 11817 11499 97.3 7.0 8355.84 12.84 0.067 0.043

PLAT056_ALERT_1_A Crystal Radius Missing for Spherical Correction Please Do !

Alert level B

PLAT043_ALERT_1_B Calculated and Reported Mol. Weight Differ by .. 34.23 Check
PLAT306_ALERT_2_B Isolated Oxygen Atom (H-atoms Missing ?) .... O90 Check

Author Response: These alerts are related to missing hydrogen atoms coming from water molecules. These atoms could not be located due to low quality of the electron density maps.

PLAT306_ALERT_2_B Isolated Oxygen Atom (H-atoms Missing ?) .... O93 Check

Author Response: These alerts are related to missing hydrogen atoms coming from water molecules. These atoms could not be located due to low quality of the electron density maps.

PLAT306_ALERT_2_B Isolated Oxygen Atom (H-atoms Missing ?) .... O94 Check

Author Response: These alerts are related to missing hydrogen atoms coming from water molecules. These atoms could not be located due to low quality of the electron density maps.

```
PLAT306_ALERT_2_B Isolated Oxygen Atom (H-atoms Missing ?) .......        O95 Check
```

Author Response: These alerts are related to missing hydrogen atoms coming from water molecules. These atoms could not be located due to low quality of the electron density maps.

```
PLAT430_ALERT_2_B Short Inter D...A Contact  O42      ..O90      .       2.79 Ang.
                                                    x,y,z  =      1_555 Check
```

Author Response: These alerts are related to missing hydrogen atoms coming from water molecules. Hence, intermolecular hydrogen atoms cannot be observed and short distances are reported.

```
PLAT430_ALERT_2_B Short Inter D...A Contact  O44      ..O94      .       2.73 Ang.
                                                  1+x,y,z  =      1_655 Check
```

Author Response: These alerts are related to missing hydrogen atoms coming from water molecules. Hence, intermolecular hydrogen atoms cannot be observed and short distances are reported.

```
PLAT430_ALERT_2_B Short Inter D...A Contact  O64      ..O93      .       2.73 Ang.
                                                    x,y,z  =      1_555 Check
```

Author Response: These alerts are related to missing hydrogen atoms coming from water molecules. Hence, intermolecular hydrogen atoms cannot be observed and short distances are reported.

```
PLAT430_ALERT_2_B Short Inter D...A Contact  O64      ..O107     .       2.78 Ang.
                                                 x,y,-1+z  =      1_554 Check
```

Author Response: These alerts are related to missing hydrogen atoms coming from water molecules. Hence, intermolecular hydrogen atoms cannot be observed and short distances are reported.

```
PLAT430_ALERT_2_B Short Inter D...A Contact  O90      ..O102     .       2.63 Ang.
                                                  1+x,y,z  =      1_655 Check
```

Author Response: These alerts are related to missing hydrogen atoms coming from water molecules. Hence, intermolecular hydrogen atoms cannot be observed and short distances are reported.

```
PLAT430_ALERT_2_B Short Inter D...A Contact  O90      ..O106     .       2.68 Ang.
                                                 1+x,y,-1+z  =      1_654 Check
```

Author Response: These alerts are related to missing hydrogen atoms coming from water molecules. Hence, intermolecular hydrogen atoms cannot be observed and short distances are reported.

```
PLAT430_ALERT_2_B Short Inter D...A Contact  O90      ..O92      .       2.71 Ang.
                                                    1+x,y,z  =      1_655 Check
```

Author Response: These alerts are related to missing hydrogen atoms coming from water molecules. Hence, intermolecular hydrogen atoms cannot be observed and short distances are reported.

```
PLAT430_ALERT_2_B Short Inter D...A Contact  O90      ..O96      .       2.73 Ang.
                                                 1+x,y,-1+z  =      1_654 Check
```

Author Response: These alerts are related to missing hydrogen atoms coming from water molecules. Hence, intermolecular hydrogen atoms cannot be observed and short distances are reported.

```
PLAT430_ALERT_2_B Short Inter D...A Contact  O93      ..O95      .       2.82 Ang.
                                                   x,y,-1+z  =      1_554 Check
```

Author Response: These alerts are related to missing hydrogen atoms coming from water molecules. Hence, intermolecular hydrogen atoms cannot be observed and short distances are reported.

```
PLAT430_ALERT_2_B Short Inter D...A Contact  O94      ..O95      .       2.69 Ang.
                                                      x,y,z  =      1_555 Check
```

Author Response: These alerts are related to missing hydrogen atoms coming from water molecules. Hence, intermolecular hydrogen atoms cannot be observed and short distances are reported.

```
PLAT430_ALERT_2_B Short Inter D...A Contact  O95      ..O107     .       2.55 Ang.
                                                      x,y,z  =      1_555 Check
```

Author Response: These alerts are related to missing hydrogen atoms coming from water molecules. Hence, intermolecular hydrogen atoms cannot be observed and short distances are reported.

```
PLAT430_ALERT_2_B Short Inter D...A Contact  O95      ..O97      .       2.68 Ang.
                                                      x,y,z  =      1_555 Check
```

Author Response: These alerts are related to missing hydrogen atoms coming from water molecules. Hence, intermolecular hydrogen atoms cannot be observed and short distances are reported.

Alert level C

```
CRYSC01_ALERT_1_C The word below has not been recognised as a standard
            identifier.
            salmon
CRYSC01_ALERT_1_C No recognised colour has been given for crystal colour.
PLAT041_ALERT_1_C Calc. and Reported SumFormula    Strings  Differ     Please Check
PLAT051_ALERT_1_C Mu(calc) and Mu(CIF) Ratio Differs from 1.0 by .       3.64 %
PLAT068_ALERT_1_C Reported F000 Differs from Calcd (or Missing)...     Please Check
PLAT088_ALERT_3_C Poor Data / Parameter Ratio ...................       9.90 Note
PLAT213_ALERT_2_C Atom C73             has ADP max/min Ratio .....       3.4 oblate
PLAT220_ALERT_2_C NonSolvent   Resd 1  C   Ueq(max)/Ueq(min) Range       3.4 Ratio
PLAT260_ALERT_2_C Large Average Ueq of Residue Including      O95      0.124 Check
PLAT260_ALERT_2_C Large Average Ueq of Residue Including      O98      0.106 Check
PLAT260_ALERT_2_C Large Average Ueq of Residue Including     O106      0.123 Check
PLAT260_ALERT_2_C Large Average Ueq of Residue Including     O107      0.158 Check
PLAT430_ALERT_2_C Short Inter D...A Contact  O64      ..O91      .       2.89 Ang.
                                                    x,y,z  =      1_555 Check
```

Author Response: These alerts are related to missing hydrogen atoms coming from water molecules. Hence, intermolecular hydrogen atoms cannot be observed and short distances are reported.

```
PLAT910_ALERT_3_C Missing # of FCF Reflection(s) Below Theta(Min).          7 Note
PLAT911_ALERT_3_C Missing FCF Refl Between Thmin & STh/L=    0.600        188 Report
PLAT975_ALERT_2_C Check Calcd Resid. Dens.  1.09Ang From O95     .       0.65 eA-3
PLAT975_ALERT_2_C Check Calcd Resid. Dens.  0.88Ang From O90     .       0.50 eA-3
PLAT975_ALERT_2_C Check Calcd Resid. Dens.  0.88Ang From O97     .       0.46 eA-3
PLAT975_ALERT_2_C Check Calcd Resid. Dens.  0.80Ang From O96     .       0.44 eA-3
PLAT976_ALERT_2_C Check Calcd Resid. Dens.  0.45Ang From O94     .      -0.63 eA-3
PLAT976_ALERT_2_C Check Calcd Resid. Dens.  0.74Ang From O94     .      -0.49 eA-3
PLAT976_ALERT_2_C Check Calcd Resid. Dens.  0.81Ang From O94     .      -0.47 eA-3
PLAT976_ALERT_2_C Check Calcd Resid. Dens.  0.89Ang From O93     .      -0.43 eA-3
PLAT976_ALERT_2_C Check Calcd Resid. Dens.  0.77Ang From O95     .      -0.42 eA-3
PLAT976_ALERT_2_C Check Calcd Resid. Dens.  0.41Ang From O107    .      -0.41 eA-3
```

Alert level G

```
FORMU01_ALERT_2_G  There is a discrepancy between the atom counts in the
            _chemical_formula_sum and the formula from the _atom_site* data.
            Atom count from _chemical_formula_sum:C50 H49 Co1 Cu2 Fe1 N8 O28.5
            Atom count from the _atom_site data:  C50 H32 Co1 Cu2 Fe1 N8 O28.5
ABSMU01_ALERT_1_G  Calculation of _exptl_absorpt_correction_mu
                not performed for this radiation type.
CELLZ01_ALERT_1_G Difference between formula and atom_site contents detected.
CELLZ01_ALERT_1_G WARNING: H atoms missing from atom site list. Is this intentional?
           From the CIF: _cell_formula_units_Z    4
           From the CIF: _chemical_formula_sum  C50 H49 Co Cu2 Fe N8 O28.50
```

```
          TEST: Compare cell contents of formula and atom_site data

          atom    Z*formula  cif sites diff
          C        200.00    200.00    0.00
          H        196.00    128.00   68.00
          Co         4.00      4.00    0.00
          Cu         8.00      8.00    0.00
          Fe         4.00      4.00    0.00
          N         32.00     32.00    0.00
          O        114.00    114.00    0.00
PLAT003_ALERT_2_G Number of Uiso or Uij Restrained non-H Atoms ...          9 Report
PLAT004_ALERT_5_G Polymeric Structure Found with Maximum Dimension          1 Info
PLAT042_ALERT_1_G Calc. and Reported Moiety Formula Strings Differ     Please Check
PLAT045_ALERT_1_G Calculated and Reported Z Differ by a Factor ...     0.5000 Check
PLAT092_ALERT_4_G Check: Wavelength Given is not Cu,Ga,Mo,Ag,In Ka    0.79990 Ang.
PLAT186_ALERT_4_G The CIF-Embedded .res File Contains ISOR Records          5 Report
PLAT300_ALERT_4_G Atom Site Occupancy of O91        Constrained at        0.6 Check
PLAT300_ALERT_4_G Atom Site Occupancy of O92        Constrained at        0.6 Check
PLAT300_ALERT_4_G Atom Site Occupancy of O96        Constrained at        0.6 Check
PLAT300_ALERT_4_G Atom Site Occupancy of O97        Constrained at       0.35 Check
PLAT300_ALERT_4_G Atom Site Occupancy of O98        Constrained at        0.4 Check
PLAT300_ALERT_4_G Atom Site Occupancy of O99        Constrained at       0.25 Check
PLAT300_ALERT_4_G Atom Site Occupancy of O101       Constrained at        0.4 Check
PLAT300_ALERT_4_G Atom Site Occupancy of O102       Constrained at        0.4 Check
PLAT300_ALERT_4_G Atom Site Occupancy of O106       Constrained at        0.4 Check
PLAT300_ALERT_4_G Atom Site Occupancy of O107       Constrained at       0.25 Check
PLAT300_ALERT_4_G Atom Site Occupancy of O109       Constrained at       0.25 Check
PLAT302_ALERT_4_G Anion/Solvent/Minor-Residue Disorder (Resd  4  )       100% Note
PLAT302_ALERT_4_G Anion/Solvent/Minor-Residue Disorder (Resd  5  )       100% Note
PLAT302_ALERT_4_G Anion/Solvent/Minor-Residue Disorder (Resd  9  )       100% Note
PLAT302_ALERT_4_G Anion/Solvent/Minor-Residue Disorder (Resd 10  )       100% Note
PLAT302_ALERT_4_G Anion/Solvent/Minor-Residue Disorder (Resd 11  )       100% Note
PLAT302_ALERT_4_G Anion/Solvent/Minor-Residue Disorder (Resd 12  )       100% Note
PLAT302_ALERT_4_G Anion/Solvent/Minor-Residue Disorder (Resd 13  )       100% Note
PLAT302_ALERT_4_G Anion/Solvent/Minor-Residue Disorder (Resd 14  )       100% Note
PLAT302_ALERT_4_G Anion/Solvent/Minor-Residue Disorder (Resd 15  )       100% Note
PLAT302_ALERT_4_G Anion/Solvent/Minor-Residue Disorder (Resd 16  )       100% Note
PLAT302_ALERT_4_G Anion/Solvent/Minor-Residue Disorder (Resd 17  )       100% Note
PLAT311_ALERT_2_G Isolated Disordered Oxygen Atom (No H's ?) .....        O91 Check
PLAT311_ALERT_2_G Isolated Disordered Oxygen Atom (No H's ?) .....        O92 Check
PLAT311_ALERT_2_G Isolated Disordered Oxygen Atom (No H's ?) .....        O96 Check
PLAT311_ALERT_2_G Isolated Disordered Oxygen Atom (No H's ?) .....        O97 Check
PLAT311_ALERT_2_G Isolated Disordered Oxygen Atom (No H's ?) .....        O98 Check
PLAT311_ALERT_2_G Isolated Disordered Oxygen Atom (No H's ?) .....        O99 Check
PLAT311_ALERT_2_G Isolated Disordered Oxygen Atom (No H's ?) .....       O101 Check
PLAT311_ALERT_2_G Isolated Disordered Oxygen Atom (No H's ?) .....       O102 Check
PLAT311_ALERT_2_G Isolated Disordered Oxygen Atom (No H's ?) .....       O106 Check
PLAT311_ALERT_2_G Isolated Disordered Oxygen Atom (No H's ?) .....       O107 Check
PLAT311_ALERT_2_G Isolated Disordered Oxygen Atom (No H's ?) .....       O109 Check
PLAT432_ALERT_2_G Short Inter X...Y Contact  O82      ..C31              2.98 Ang.
                                             x,1/2-y,-1/2+z  =      4_565 Check
PLAT432_ALERT_2_G Short Inter X...Y Contact  O84      ..C30              2.91 Ang.
                                              x,1/2-y,1/2+z  =      4_566 Check
PLAT432_ALERT_2_G Short Inter X...Y Contact  O93      ..C21              2.85 Ang.
                                                   -1+x,y,z  =      1_455 Check
PLAT432_ALERT_2_G Short Inter X...Y Contact  O94      ..C40              2.97 Ang.
                                                 -1+x,y,1+z  =      1_456 Check
```

```
PLAT432_ALERT_2_G Short Inter X...Y Contact  O99      ..C19             2.97 Ang.
                                                    x,y,1+z  =      1_556 Check
PLAT650_ALERT_4_G SWAT Instruction Used to Model Solvent Disorder          ! Report
PLAT794_ALERT_5_G Tentative Bond Valency for Cu1       (II)      .       2.22 Info
PLAT794_ALERT_5_G Tentative Bond Valency for Cu2       (II)      .       2.13 Info
PLAT794_ALERT_5_G Tentative Bond Valency for Co3       (III)     .       3.32 Info
PLAT794_ALERT_5_G Tentative Bond Valency for Fe4       (III)     .       3.04 Info
PLAT860_ALERT_3_G Number of Least-Squares Restraints .............         54 Note
PLAT883_ALERT_1_G No Info/Value for _atom_sites_solution_primary .     Please Do !
PLAT898_ALERT_4_G Second Reported H-M Symbol in CIF Ignored ......          ! Check
PLAT912_ALERT_4_G Missing # of FCF Reflections Above STh/L=  0.600         68 Note
PLAT913_ALERT_3_G Missing # of Very Strong Reflections in FCF ....          2 Note
PLAT933_ALERT_2_G Number of HKL-OMIT Records in Embedded .res File          1 Note
PLAT952_ALERT_5_G Calculated (ThMax) and CIF-Reported Lmax Differ           4 Units
PLAT958_ALERT_1_G Calculated (ThMax) and Actual (FCF) Lmax Differ           4 Units
PLAT965_ALERT_2_G The SHELXL WEIGHT Optimisation has not Converged     Please Check
PLAT978_ALERT_2_G Number C-C Bonds with Positive Residual Density.          2 Info
PLAT984_ALERT_1_G The Co-f'=    0.3450 Deviates from the B&C-Value     0.3429 Check
PLAT984_ALERT_1_G The Cu-f'=    0.2660 Deviates from the B&C-Value     0.2701 Check
PLAT984_ALERT_1_G The Fe-f'=    0.3580 Deviates from the B&C-Value     0.3563 Check
PLAT985_ALERT_1_G The Cu-f"=    1.5610 Deviates from the B&C-Value     1.5646 Check
PLAT985_ALERT_1_G The Fe-f"=    1.0500 Deviates from the B&C-Value     1.0510 Check
```

```
   2 ALERT level A = Most likely a serious problem - resolve or explain
  17 ALERT level B = A potentially serious problem, consider carefully
  25 ALERT level C = Check. Ensure it is not caused by an omission or oversight
  68 ALERT level G = General information/check it is not something unexpected

  19 ALERT type 1 CIF construction/syntax error, inconsistent or missing data
  54 ALERT type 2 Indicator that the structure model may be wrong or deficient
   6 ALERT type 3 Indicator that the structure quality may be low
  27 ALERT type 4 Improvement, methodology, query or suggestion
   6 ALERT type 5 Informative message, check
```

It is advisable to attempt to resolve as many as possible of the alerts in all categories. Often the minor alerts point to easily fixed oversights, errors and omissions in your CIF or refinement strategy, so attention to these fine details can be worthwhile. In order to resolve some of the more serious problems it may be necessary to carry out additional measurements or structure refinements. However, the purpose of your study may justify the reported deviations and the more serious of these should normally be commented upon in the discussion or experimental section of a paper or in the "special_details" fields of the CIF. checkCIF was carefully designed to identify outliers and unusual parameters, but every test has its limitations and alerts that are not important in a particular case may appear. Conversely, the absence of alerts does not guarantee there are no aspects of the results needing attention. It is up to the individual to critically assess their own results and, if necessary, seek expert advice.

Publication of your CIF in IUCr journals

A basic structural check has been run on your CIF. These basic checks will be run on all CIFs submitted for publication in IUCr journals (*Acta Crystallographica*, *Journal of Applied Crystallography*, *Journal of Synchrotron Radiation*); however, if you intend to submit to *Acta Crystallographica Section C* or *E* or *IUCrData*, you should make sure that full publication checks are run on the final version of your CIF prior to submission.

Publication of your CIF in other journals

Please refer to the *Notes for Authors* of the relevant journal for any special instructions relating to CIF submission.

PLATON version of 18/12/2021; check.def file version of 18/12/2021

Datablock be2-auto-sphere - ellipsoid plot

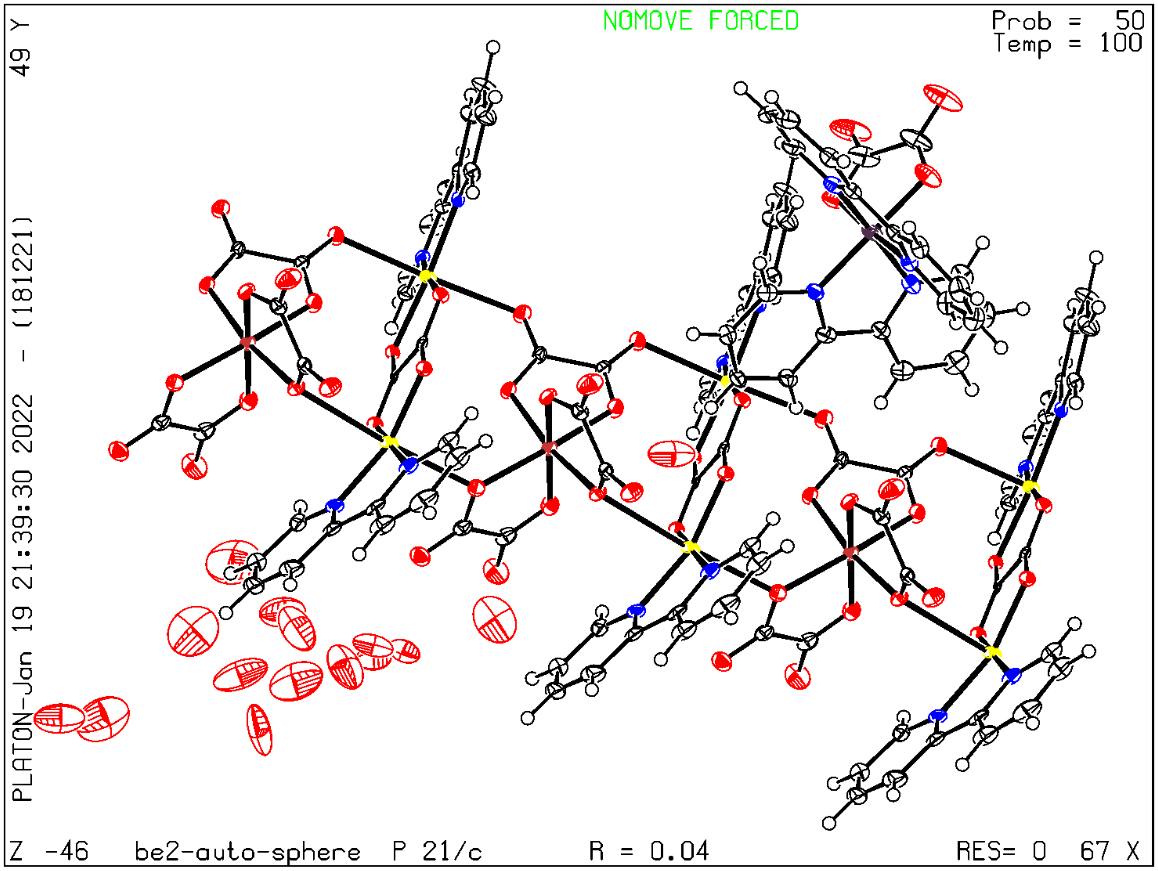

Supplement: Supplementary file 1 [file ijms-23-01556-s001.zip › be2-auto-sphere-11f2-revised-final-checkcif.pdf]
