# Supplementary material for: Sorption and Magnetic Properties of Oxalato-Based Trimetallic Open Framework Stabilized by Charge-Assisted Hydrogen Bonds
Source: Int J Mol Sci. 2022 Jan 29;23(3):1556. doi: 10.3390/ijms23031556 (PMC8835875; doi:10.3390/ijms23031556)
Supplement: Supplementary file 1 [file ijms-23-01556-s001.zip › muziol-Supplementary-Materials.pdf]

Magnetic and sorption properties of oxalato based trimetallic open framework stabilized by charge-assisted hydrogen bonds.

Tadeusz M. Muzioł*[a], Natalia Tereba[a], Robert Podgajny[b], Robert Pełka[c], Dominika Czernia[c], Marek Wiśniewski[a], Stanisław Koter[a], Grzegorz Wrzeszcz[a],

[a] Faculty of Chemistry, Nicolaus Copernicus University in Toruń, Gagarina 7, 87-100 Toruń, Poland.
[b] Faculty of Chemistry, Jagiellonian University, Gronostajowa 2, 30-387 Kraków, Poland.
[c] Institute of Nuclear Physics of Polish Academy of Sciences, Radzikowskiego 152, 31-342 Kraków, Poland.
* corresponding author

Supplementary Materials

Contents

Figures S3
Figure S1. The coordination units in [Co(bpy)$_2$(ox)][{Cu$_2$(bpy)$_2$(ox)}Fe(ox)$_3$]·8.5H$_2$O. S3
Figure S2. The four oxalate coordination modes in **NCU-1.** S3
Figure S3. Distance d(Cu-Cu). S4
Figure S4. Comparison of the elongated Cu-O bonds. S4
Figure S5. Superposition of **NCU-1** with ammonium and potassium complexes. S5
Figure S6. Thermal analysis of [Co(bpy)$_2$(ox)][{Cu$_2$(bpy)$_2$(ox)}Fe(ox)$_3$]·8.5H$_2$O **(NCU-1).** S5
Figure S7. Comparison of powder diffractograms. S6
Figure S8. Changes in band intensity during H$_2$O/D$_2$O exchange. S6
Tables S7
Table S1. Crystal data and structure refinement for **NCU-1.** S7
Table S2. Comparison of structural and magnetic properties for selected compounds. S8
Table S3. Selected bond distances and valence angles for **NCU-1.** S11
Table S4. Intermolecular hydrogen bonds in **NCU-1**. S12
XAS S13
Table S5. Summary of Fe, Co and Cu L-edges for **NCU-1**. S13
Figure S9. Normalized XANES spectra of iron and copper $L_{2,3}$ edges. S14

Structural analysis of $[Cu_2(AA)_2(ox)]^{2+}$ motifs (AA = bpy or other N-donor ligand). S15
Figure S10. Selected parameters describing the oxalate bridge geometry. S16
Figure S11. Angle between copper basal planes. S18
Figure S12. Plot of Cu shift from the copper base. S18
Figure S13. Angles between imine moieties. S19
Figure S14. Distance between bpy centroids *vs*. the average value of bpy/ox plane. S19
Computational details of the MF approach S20
References S22

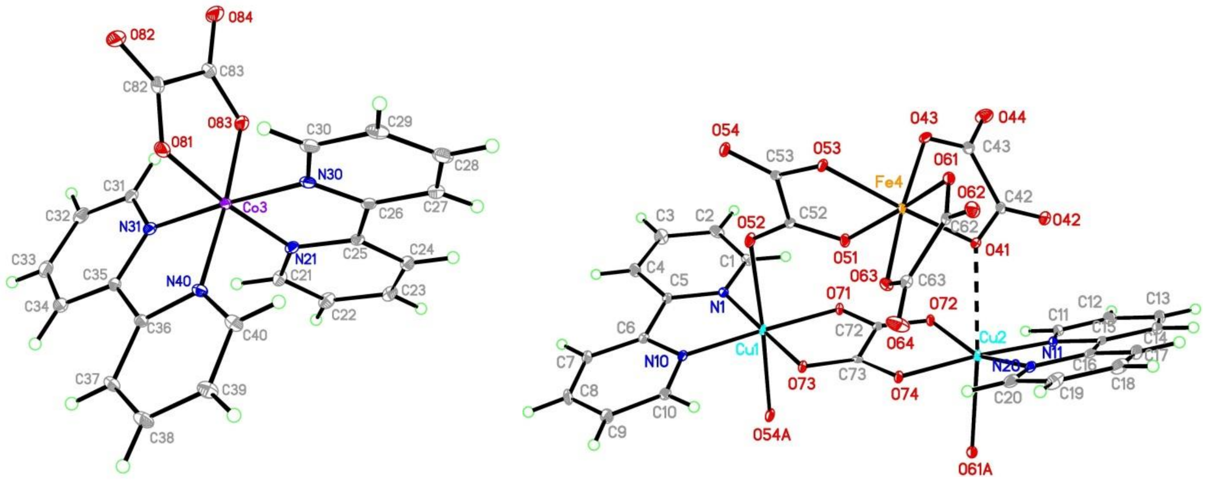

Figure S1. The $[Co(bpy)_2(ox)]^+$ (left) and $[\{Cu_2(bpy)_2ox\}Fe(ox)_3]^-$ (right) units are presented with thermal ellipsoids at 30% probability for the clarity of the picture. Crystallization water molecules are skipped.

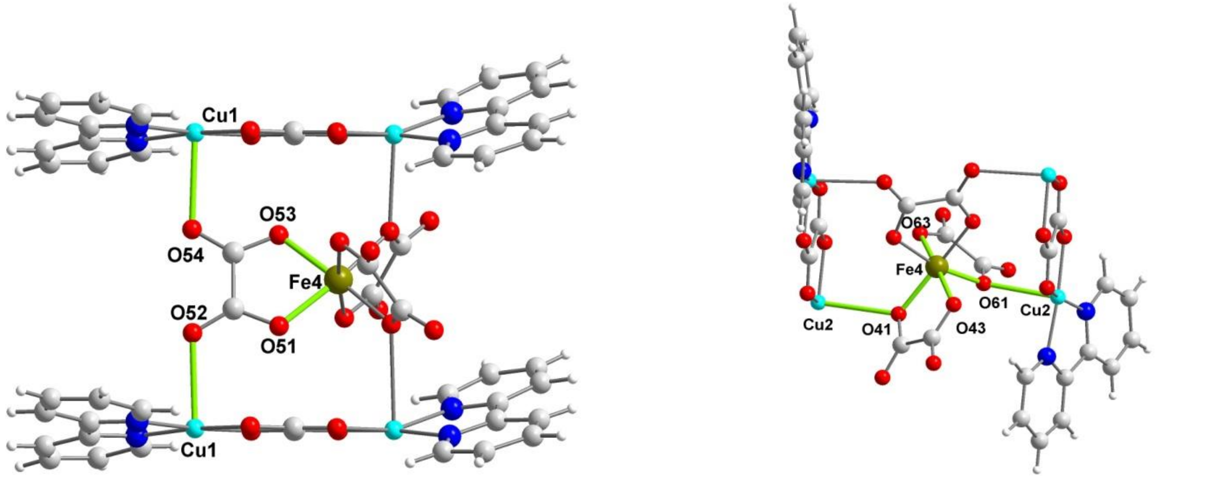

(a) (b)

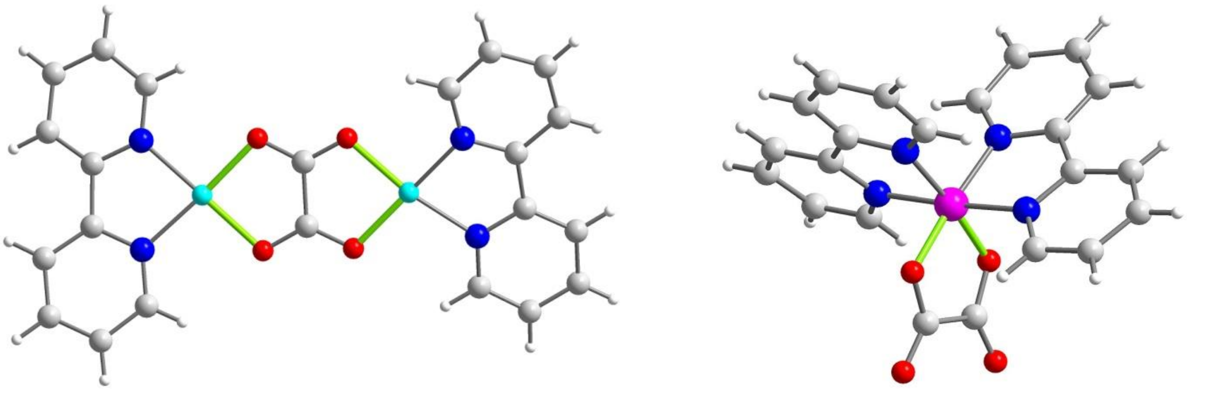

(c) (d)

Figure S2. The four oxalate coordination modes identified in $[Co(bpy)_2(ox)][\{Cu_2(bpy)_2ox\}Fe(ox)_3]\cdot 8.5H_2O$ with Cu-O bonds involved in coordination marked in green: (a) bidentate/bismonodentate mode 1, (b) bidentate/monodentate mode 2, (c) bisbidentate mode 3, (a) bidentate mode 4.

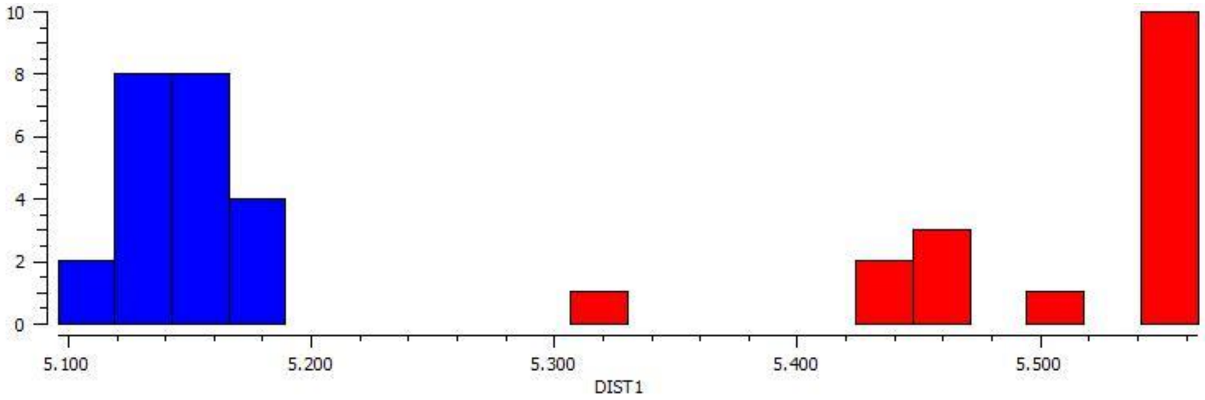

Figure S3. Distance d(Cu-Cu) with short distance in blue and long in red.

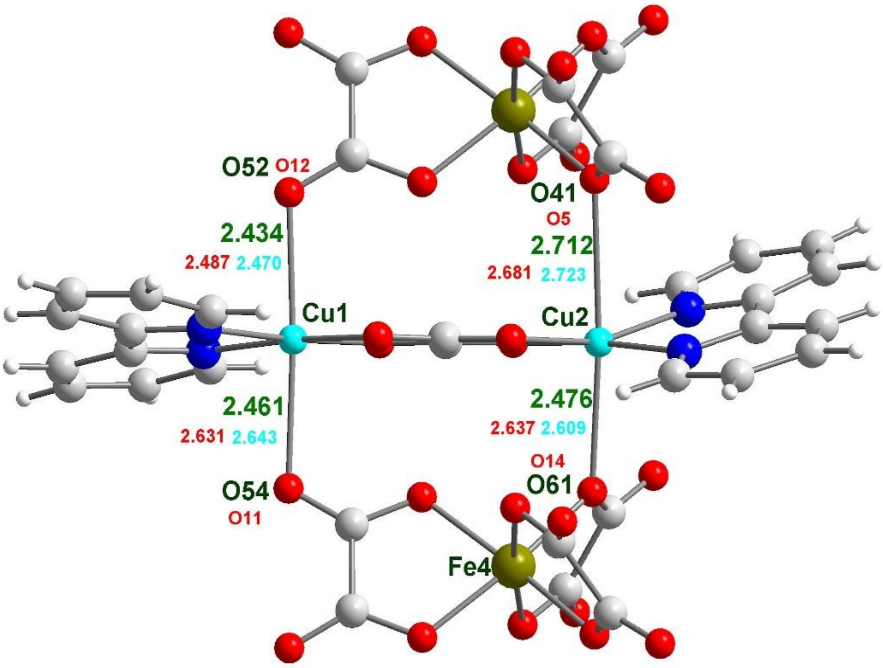

Figure S4. Comparison of the elongated Cu-O bonds in the reported complex (green) and in the ammonium (red) and potassium (cyan) compounds [1]. The atom numbering in both ammonium and potassium complexes are the same. Hence, only atom numbering for ammonium is given (atom labels in red).

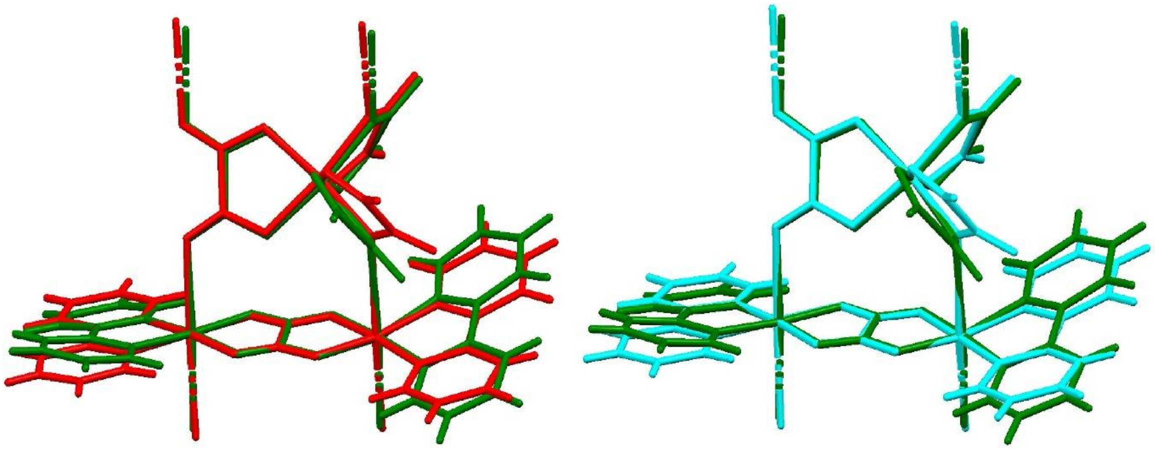

Figure S5. Superposition of the reported complex (green) with ammonium (red, left) and potassium (cyan, right) compounds [1]. All atoms from the chain $[\{Cu_2(bpy)_2(ox)\}Fe(ox)_3]^-$ were used as a pattern for superposition. These pictures present that there are significant differences between **NCU-1** and the reported motifs. The biggest differences were found for both bpy ligands due to their significant twist in **NCU-1** and O41/O5 oxalate anions with rmsd being 0.4001 and 0.3987 Å for ammonium and potassium complexes, respectively. In the reported structure, all bpy rings are flat with rmsd being 0.094, 0.027, 0.035, and 0.025 Å for N1, N11, N21 and, N31 bpy molecules, respectively. The significantly bigger deformation of N1 ligand is related to the bigger tilting of both pyridine rings (11.06 °). The basal planes of Cu(II) form angles of 7.58 (Cu1) and 14.01 °(Cu2) with bpy ligands.

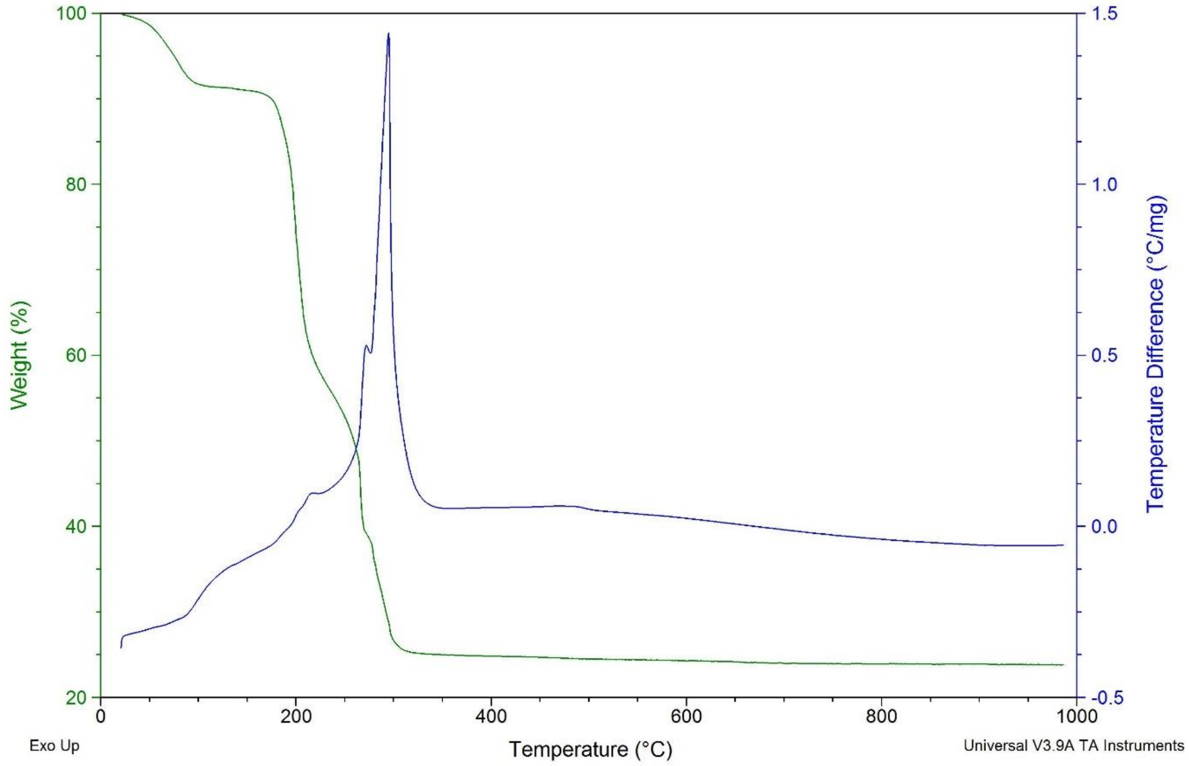

Figure S6. Thermal analysis of **NCU-1** clearly shown that the loss of crystallization water molecules is accomplished at *ca*. 120 °C.

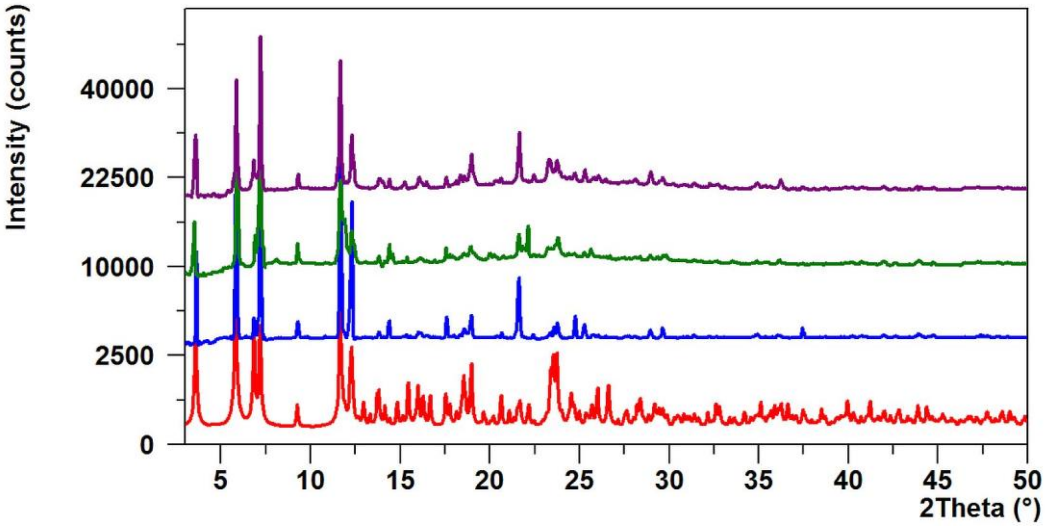

Figure S7. Powder diffractograms of the calculated pattern (red) and registered in the range 3-50 ° at room temperature for sample before heating (blue), sample after heating at 120 ℃ during 3 h (green) and sample after cooling to room temperature (violet).

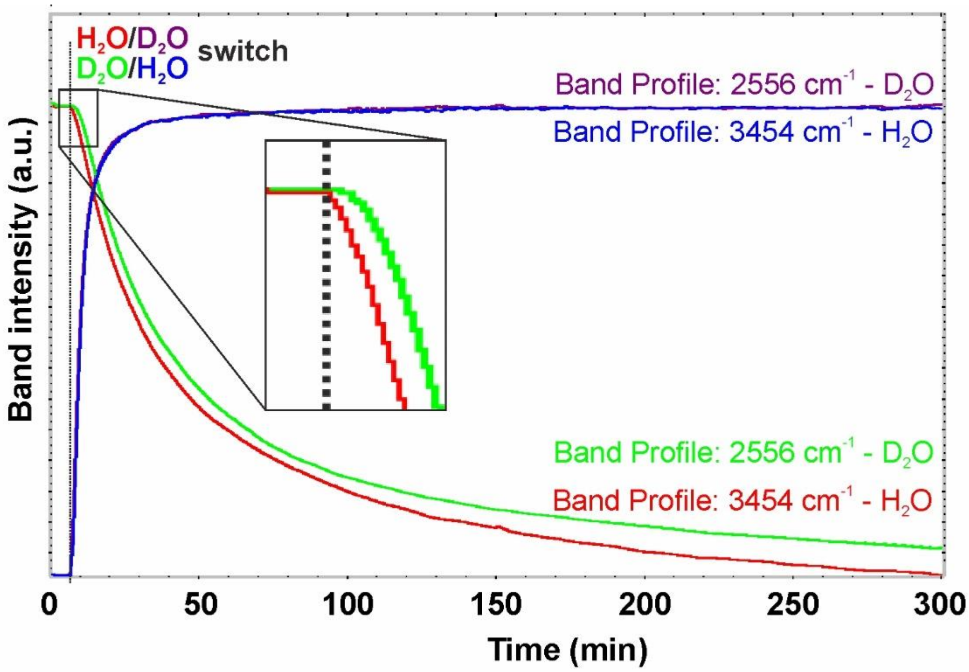

Figure S8. Changes in band intensity during H_2O/D_2O exchange. (Blue and purple adsorption; red and green desorption).

Table S1. Crystal data and structure refinement for **NCU-1.**

Identification code	**NCU-1**
Empirical formula	$C_{50} H_{49} Co Cu_2 Fe N_8 O_{28.50}$
Formula weight	1459.83
Temperature [K]	100(2)
Wavelength [Å]	0.7999
Crystal system, space group	Monoclinic, $P2_1/c$ (no 14)
Unit cell dimensions [Å] and [°]	a = 15.4103(3) b = 49.1416(9)<br>β = 100.402(2)<br>c = 7.6343(2)
Volume [$Å^3$]	5686.4(2)
Z, Calculated density [$Mg·m^{-3}$]	4, 1.705
Absorption coefficient [mm^{-1}]	1.823
F(000)	2976
Crystal size [mm]	0.200x0.070x0.050
Theta range for data collection [°]	2.780 to 29.996
Limiting indices	-19<=h<=19<br>-61<=k<=61<br>-5<=l<=5
Reflections collected/unique	64870 / 8676 [R(int) = 0.0625]
Completeness [%] to theta [°]	28.681 ° 77.4%
Max. and min. transmission	0.825 and 0.683
Data/restraints/parameters	8676 / 54 / 876
Goodness-of-fit on F^2	1.056
Final R Indices [I>2sigma(I)]	$R1^a$ = 0.0406, $wR2^b$ = 0. 1152
R indices (all data)	$R1^a$ = 0.0470, $wR2^b$ = 0.1216
Largest diff. peak and hole [$eÅ^{-3}$]	0.945 and -0.625

^{a} $R1 = \Sigma||F_o|-|F_c||/\Sigma|F_o|$ ^{b} $wR2 = [\Sigma w(F_o^2 - F_c^2)^2/\Sigma(w(F_o^2)^2)]^{1/2}$

Table S2. Selected geometrical parameters and magnetic data for compounds with $[(L)Cu(\mu\text{-}ox)Cu(L)]^{2+}$ motif (L – imine ligand with bpy skeleton).

No.	Formula	C.N.[a]	Auxiliary ligand	motif	Tetragonality	∠Cu1/Cu2	∠Cu1/ox	∠Cu2/ox	d(Cu-Cu)	d(Cu1-basalCu1)	d(Cu2-basalCu2)	J [cm^{-1}][b]	Refs.
1	$\{[Co(bpy)_2(ox)][\{Cu_2(bpy)_2(ox)\}Fe(ox)_3]\}_n \cdot 8.5nH_2O$	6	bpy	ladder	0.808<br>0.760	12.57	3.40	9.54	5.132	0.004	0.112	-275<br>-3.8	this work
2	$\{NH_4[\{Cu(bpy)\}_2(ox)Fe(ox)_3]\cdot H_2O\}_n$	6	bpy	ladder	0.776<br>0.743	8.25	2.19	7.16	5.142	0.042	0.063	-342	[1]
3	$\{K[\{Cu(bpy)\}_2(ox)Fe(ox)_3]\cdot H_2O\}_n$	6	bpy	ladder<br>→ 2D	0.772<br>0.739	7.12	2.60	6.32	5.140	0.042	0.080	-227	[1]
4	$\{NH_4[CrCu_2(bpy)_2(ox)_4]\cdot H_2O\}_n$	6	bpy	ladder	0.783<br>0.735	7.12	6.24	2.01	5.135	0.071	0.031	-371	[2]
5	$\{K[CrCu_2(bpy)_2(ox)_4]\cdot H_2O\}_n$	6	bpy	ladder<br>→ 2D	0.737<br>0.784	6.28	5.89	2.81	5.139	0.092	0.032	-343	[2]
6	$[(Cr_2O_7)Cu_2(ox)(phen)_2]_n$	6	bpy	ladder	0.826	0.00	12.53	12.53	5.132	0.172	0.172	-340[c]	[2]
7	$[\{Cu(phen)_2\}_2(ox)][Cr(phen)(ox)_2]_2\cdot 4H_2O$	*6	bpy	dimer	0.908	0.00	87.28	87.28	5.567	0.044	0.044	+3.45	[3]
8	$[Cu_2(bpy)_2(H_2O)_2(ox)](CF_3SO_3)_2$	6	bpy	dimer	0.783	0.00	1.14	1.14	5.127	0.041	0.041	-384	[4]
9	$[Cu_2(bpy)_2(ox)](ClO_4)_2$	6	bpy	ladder	0.783<br>0.794	1.94	10.53	11.79	5.152	0.155	0.167	-387	[4]
10	$[Cu_2(bpy)_2(ox)](PF_6)_2$	5+1	bpy	ladder	0.807	0.00	3.29	3.29	5.131	0.079	0.079	-392	[4]
11	$[Cu_2(bpy)_2Cl_2(ox)]\cdot H_2O$	5	bpy	dimer	0.811	29.66	14.83	14.83	5.1846	0.367	0.367	-328	[4]
12	$[Cu_2(bpy)_2(NO_2)_2(ox)]$	*5+1	bpy	dimer	0.830	0.00	82.69	82.69	5.460	0.127	0.127	-14	[4]
13	$[Cu(bpy)(ox)]\cdot 2H_2O$	6	bpy	1D	0.863	0.00	83.43	83.43	5.161	0.034	0.034	+2.44	[5]
14	$[\{Cu(NO_3)(H_2O)(bpy)\}_2(ox)]$	6	bpy	dimer	0.774	0.00	4.59	4.59	5.143	0.112	0.112	-382	[6]
15	$[Cr_2Cu_2(bpy)_4(ox)_5]\cdot 2H_2O$	*6	bpy	tetramer	0.871	0.00	81.68	81.68	5.438	0.031	0.031	+4	[7]
16	$[Cu_2(bpy)_2(H_2O)_2(ox)](NO_3)_2\cdot[Cu(bpy)(ox)]$	5	bpy	dimer	0.882	0.00	3.18	3.18	5.154	0.155	0.155	-386	[8]
17	$[Cu_2(bpy)_2(H_2O)_2(ox)](BF_4)_2\cdot[Cu(bpy)(ox)]$	5	bpy	dimer	0.868	0.0	10.43	10.43	5.144	0.165	0.165	-378	[8]
18	$[Cu_2(bpy)_2(H_2O)_2(ox)](ClO_4)_2\cdot[Cu(bpy)(ox)]$	5	bpy	dimer	0.869	0.0	12.06	12.06	5.150	0.180	0.180	-376	[8]

19	$[Cu(bpy)(ox)]_n$	*6	bpy	1D	0.861<br>0.860	0.45	83.97	83.52	5.548	0.038	0.032	ND	[9]
20	$[\{Cu(Mebpy)(NO_3)(H_2O)\}_2(ox)]$ [d]	6	4,4'-<br>Mebpy	dimer	0.814	0.00	4.39	4.39	5.161	0.005	0.005	ND	[10]
21	$[\{Cu(Mebpy)(ClO_4)(H_2O)\}_2(ox)]$ [d]	6	4,4'-<br>Mebpy	dimer	0.801	0.00	2.35	2.35	5.173	0.022	0.022	ND	[10]
22	$[Cu(ox)(bpy)]_n$	*6	bpy	1D	0.846	0.00	70.99	70.99	5.507	0.020	0.020	ND	[11]
23	$[\{Cu(phen)(CH_3CO_2)\}(ox)]$	*5+1	phen	dimer	0.820	0.00	81.15	81.15	5.436	0.130	0.130	ND	[12]
24	$[Cu_2(L)_2(ox)(phen)_2]$ [e]	*5+1	phen	dimer	0.842	0.00	70.94	70.94	5.318	0.309	0.309	ND	[13]
25	$[\{Cu(NO_3)(phen)(H_2O)\}_2(ox)]$	6	phen	dimer	0.807	0.00	5.42	5.42	5.115	0.016	0.016	ND	[14]
26	$\{[Cu_2(bpy)_2(ox)]_3[P_2W_{18}O_{62}]\}_n$ $3nH_2O$	5<br>6<br>5	bpy	1D	0.837<br>0.793<br>0.874	0.00<br>0.00<br>0.00	11.74<br>7.63<br>17.86	11.74<br>7.63<br>17.86	5.096<br>5.141<br>5.165	0.138<br>0.142<br>0.313	0.138<br>0.142<br>0.313	ND	[15]
27	$Rb_4[Cu(phen)(H_2O)_4]_2[Cu_4(phen)_4(H_2O)_4(ox)_3]_{0.6}$<br>$[Cu_2(phen)_2(H_2O)_4(ox)]_{0.4}[Cu(phen)(ox)]_{0.8}$<br>$[\{SiW_{11}O_{39}Cu(H_2O)\}_2\{Cu_2(phen)_2(ox)\}]\cdot 20H_2O$	6<br>*6	phen	1D	0.770<br>0.832	0.00<br>0.00	4.77<br>86.20	4.77<br>86.20	5.137<br>5.459	0.020<br>0.039	0.020<br>0.039	-344[f]<br>-11.1	[16]
28	$(NH_4)_4[Cu(phen)(H_2O)_4]_2[Cu_4(phen)_4(H_2O)_4(ox)_3]_{0.6}$<br>$[Cu_2(phen)_2(H_2O)_4(ox)]_{0.4}[Cu(phen)(ox)]_{0.8}$<br>$[\{SiW_{11}O_{39}Cu\text{-}(H_2O)\}_2\{Cu_2(phen)_2(ox)\}]\cdot 20H_2O$	6<br>*6	phen	1D	0.759<br>0.833	0.00<br>0.00	4.66<br>87.45	4.66<br>87.45	5.139<br>5.464	0.021<br>0.068	0.021<br>0.068	-354[f]<br>-11.8	[16]
29	$\{K_{14}[\{Cu_2(bpy)_2(ox)\}\{SiW_{11}O_{39}Cu(H_2O)\}]_2\}_n$<br>$[SiW_{11}O_{39}Cu(H_2O)]\cdot\sim 55H_2O$	5	bpy	1D→2D	0.774	0.00	0.00	0.00	5.147	0.002	0.002	-344[g]	[17]
30	$\{\{[PMo_9V_5O_{42}][Cu_2(bpy)_2(ox)]\}[Cu(bpy)_2(H_2O)]$<br>$[Cu(bpy)_2Cl]\}_n$ $3nH_2O$	6	bpy	1D	0.787<br>0.777	26.95	13.90	13.10	5.096	0.096	0.045	ND	[18]
31	$\{K_2H_7[\{Pr(PW_{11}O_{39})_2\}\{Cu_2(bpy)_2(ox)\}]\}_n\cdot 18nH_2O$	6	bpy	1D	0.794	0.00	2.19	2.19	5.158	0.055	0.055	ND[h]	[19]
32	$\{K_2H_7[\{Gd(PW_{11}O_{39})_2\}\{Cu_2(bpy)_2(ox)\}]\}_n\cdot 22nH_2O$	6	bpy	1D	0.799	0.00	2.17	2.17	5.175	0.067	0.067	ND	[19]
33	$\{K_2H_7[\{Yb(PW_{11}O_{39})_2\}\{Cu_2(bpy)_2(ox)\}]\}_n\cdot 19nH_2O$	6	bpy	1D	0.790	0.00	2.40	2.40	5.167	0.066	0.066	ND	[19]
34	$\{K_2H_9[\{K(PW_{11}O_{39})_2\}\{Cu_2(bpy)_2(ox)\}]\}_n\cdot 20.5nH_2O$	6	bpy	1D	0.789	0.00	1.01	1.01	5.180	0.030	0.030	ND	[19]
35	$\{K_2H_7[\{La(PW_{11}O_{39})_2\}\{Cu_2(bpy)_2(ox)\}]\}_n\cdot 18nH_2O$	6	bpy	1D	0.800	0.00	2.01	2.01	5.183	0.059	0.059	-347	[19]
36	$[Cu(ox)(\mu\text{-bpa})]_n$	6	bpa[i]	2D	0.869	0.00	78.99	78.99	5.496	0.000	0.000	+2.50	[20]

37	[Cu(ox)(μ-bpe)]	6	bpe[i]	2D	0.948	0.0	90	90	5.510	0.000	0.000	+2.00	[20]
38	$[Cu_2(TAcO)_2(ox)(4,4'\text{-bpy})]\cdot 4H_2O$	5	4,4'-bpy [j]	2D	0.901	0.00	1.02	1.02	5.210	0.150	0.150	-293	[21]
39	$[\{Cu(tmen)(H_2O)\}_2(ox)](ClO_4)_2\cdot 1.25H_2O$	5/ 5	tmen[k]	dimer	0.843 (Cu1) 0.863 (Cu2)	0.00/ 0.00	12.04/ 8.41	12.04/ 8.41	5.146/ 5.167	0.148/0.181	0.148/0.181	-385.4	[22]
40	$[dienCu(ox)Cu(H_2O)_2(tmen)](ClO_4)_2$	6	dien/ tmen[k]	1D	0.742 (Cu1) 0.760 (Cu2)	67.97	12.10	79.96	5.399	0.014	0.069	-75.5	[22]
41	$[\{Cu(2\text{-MeIm})tmen\}_2(ox)](PF_6)_2$	5	tmen[k]	dimer	0.914	0.00	83.41	83.41	5.434	0.207	0.207	-13.8	[22]

[a] C.N. - coordination number, *means that two oxygen atoms come from two different O donor ligands, [b] data according to the Hamiltonian in the form $H = -JS_aS_b$, some original data have been recalculated; ND – no data available, [c] from DFT calculations, [d] 4,4'-dimethyl-2,2'-bipyridine, [e] benzene-1,3,5-tricarboxylate, [f] the first value corresponds to $[Cu_2(phen)_2(ox)]^{2+}$ metalorganic block (MB) and the second to the $[Cu_2(phen)_2(H_2O)_2(ox)]^{2+}$, [g] for data fitted in the high temperature region (above 100 K), [h] the model is not given and J value is unavailable. Only θ = -172.927 K is reported, [i] bpa=1,2-bis[4-pyridyl)ethane, bpe=1,2-bis(4-pyridyl)ethylene, [j] TAcO=thymine, 4,4'-bpy= 4,4'-bipyridine, [k] tmen=*N*,*N*,*N*',*N*'-tetramethylethylenediamine, dien=diethylenetriamine, 2-MeIm=2-methylimidazole,

Table S3. Selected bond distances and valence angles for **NCU-1.**

Cu1-O71	1.9691(19)	Cu2-N11	1.965(3)
Cu1-O73	1.9787(19)	Cu2-O74	1.9691(19)
Cu1-N1	1.982(2)	Cu2-N20	1.973(3)
Cu1-N10	1.983(2)	Cu2-O72	1.9745(19)
Cu1-O52	2.434(3)	Cu2-O41	2.712(2)
Cu1-O54 ^{i}	2.461(3)	Cu2-O61 ^{i}	2.476(2)
Fe4-O41	2.005(2)	Co3-O83	1.893(2)
Fe4-O51	2.006(2)	Co3-O81	1.900(2)
Fe4-O43	2.009(2)	Co3-N31	1.947(3)
Fe4-O63	2.009(2)	Co3-N30	1.947(3)
Fe4-O53	2.009(2)	Co3-N21	1.954(3)
Fe4-O61	2.023(2)	Co3-N40	1.954(3)
O71-Cu1-O73	86.33(8)	N11-Cu2-O74	175.28(11)
O71-Cu1-N1	95.32(9)	N11-Cu2-N20	82.29(12)
O73-Cu1-N1	175.91(11)	O74-Cu2-N20	98.79(10)
O71-Cu1-N10	174.87(11)	N11-Cu2-O72	95.64(10)
O73-Cu1-N10	96.59(9)	O74-Cu2-O72	84.74(8)
N1-Cu1-N10	82.06(10)	N20-Cu2-O72	162.05(11)
O71-Cu1-O52	92.77(9)	N11-Cu2-O41	91.52(10)
O73-Cu1-O52	89.64(8)	O74-Cu2-O41	93.20(8)
N1-Cu1-O52	86.54(10)	N20-Cu2-O41	78.93(9)
N10-Cu1-O52	91.47(10)	O72-Cu2-O41	83.32(8)
O71-Cu1-O54^{i}	89.35(8)	N11-Cu2-O61^{i}	90.61(10)
O73-Cu1-O54^{i}	92.88(9)	O74-Cu2-O61^{i}	84.67(8)
N1-Cu1-O54^{i}	90.88(10)	N20-Cu2-O61^{i}	104.93(10)
N10-Cu1-O54^{i}	86.30(10)	O72-Cu2-O61^{i}	92.89(8)
O52-Cu1-O54^{i}	176.81(7)	O61^{i}-Cu2-O41	175.82(7)
O41-Fe4-O51	93.59(9)	O83-Co3-O81	87.14(11)
O41-Fe4-O43	80.93(9)	O83-Co3-N31	93.67(11)
O51-Fe4-O43	93.25(9)	O81-Co3-N31	88.37(11)
O41-Fe4-O63	95.12(9)	O83-Co3-N30	89.11(11)
O51-Fe4-O63	90.39(9)	O81-Co3-N30	93.47(11)
O43-Fe4-O63	174.78(9)	N31-Co3-N30	176.74(11)
O41-Fe4-O53	169.40(9)	O83-Co3-N21	88.47(11)
O51-Fe4-O53	80.13(10)	O81-Co3-N21	174.26(10)

O43-Fe4-O53	90.85(9)	N31-Co3-N21	95.59(12)
O63-Fe4-O53	93.46(9)	N30-Co3-N21	82.77(12)
O41-Fe4-O61	96.41(10)	O83-Co3-N40	175.41(11)
O51-Fe4-O61	167.27(9)	O81-Co3-N40	90.37(11)
O43-Fe4-O61	96.08(9)	N31-Co3-N40	82.41(12)
O63-Fe4-O61	80.90(9)	N30-Co3-N40	94.89(12)
O53-Fe4-O61	91.08(9)	N21-Co3-N40	94.26(11)

[i] (x, y, -1+z)

Table S4. Intermolecular hydrogen bonds (intramolecular bonds are omitted) responsible for crystal maintaining.

**D-H…A [operator]**	**H…A [Å]**	**D…A[Å]**	**∠ D-H…A [°]**	**Type of hydrogen bond**
C2-H2…O84 **[x, y, z]**	2.34	3.169	146	Co-chain
C4-H4…O52 [x, 1/2-y, -1/2+z]	2.60	3.367	138	Chain-chain along b
C4-H4…O54 [x, 1/2-y, -1/2+z]	2.55	3.428	154	Chain-chain along b
C7-H7…O52 [x, 1/2-y, -1/2+z]	2.54	3.423	155	Chain-chain along b
C7-H7…O54 [x, 1/2-y, -1/2+z]	2.58	3.339	137	Chain-chain along b
C9-H9…O81 [-1+x, y, z]	2.59	3.445	149	Co-chain
C9-H9…O82 [-1+x, y, z]	2.38	3.168	140	Co-chain
C13-H13…O62 [1-x,-y,1-z]	2.57	3.199	124	Chain-chain along b
C18-H18…O42 [1-x,-y,1-z]	2.40	3.252	149	Chain-chain along b
C21-H21…O93 [1+x, y, z]	2.52	2.854	101	Co-water
C24-H24…O72 [**x, y, z**]	2.52	3.177	126	Co-chain
C27-H27…O43 [x,y,-1+z]	2.47	3.359	157	Co-chain
C30-H30…O84 [x, 1/2-y, -1/2+z]	2.41	2.914	113	Co-Co
C31-H31…O82 [x, 1/2-y, 1/2+z]	2.44	2.981	116	Co-Co
C34-H34…O63 [1+x, y, z]	2.50	3.399	157	Co-chain
C37-H37…O74 [1+x, y, z]	2.56	3.186	124	Co-chain

XAS

The energy ranges for iron, cobalt, and copper were carefully selected to avoid the edge regions of other elements, what is crucial for proper spectra normalization. It is especially important for iron which has to be truncated relatively early because of the presence of cobalt. These spectra show a more intense bands corresponding to L3 edge followed by much smaller L2 peaks and the former one will be discussed in detail (Table S5). For the iron L3 edge, there are two peaks occurring at 708.3 and 710.0 eV. Similarly to $K_3[Fe(ox)_3]$ and contrary to $[Cu_2(bpy)_4Fe(ox)_3](NO_3)\cdot H_2O$ more intense is the high energy peak [23,24] (Figure 6). The energy difference is 1.7 eV and is consistent with 1.6 eV reported for $K_3[Fe(ox)_3]$ [23]. Both multiplet components are not pure t_{2g} (low energy) and e_g, (high energy) and are influenced also by other factors, e.g. π donation ability of ligands affecting mainly the low energy peak. In the reported compound, there are relatively short Fe-O bonds and C-O bonds ranging from 1.217(4) to 1.295(4) Å. Therefore, the relative intensity of both peaks indicates that only some π donation is present in **NCU-1** and some electron density delocalization over the ligand occurs. Copper(II) shows a pattern (Figure S5, Table S5) with only one peak as it is expected due to $3d^9$ configuration [25]. From the structural data with four short equatorial bonds and two long apical distances (Figure 2), we can suppose that the unpaired electron is located on the d_{x2-y2} orbital.

Table S5. Summary of spectral features of the Fe, Co and Cu absorption at the L-edges for **NCU-1**.

Element and edge	Energy peak for L3 [eV]	Maximum of intensity for L3	Energy peak for L2 [eV]	Maximum of intensity for L2
Fe L-edge	708.3, 710.0	8.80, 11.51	719.7(sh), 721.5, 723.2	1.44, 2.91, 2.59
Co L-edge	777.2, 779.0, 781.0, 783.7	1.70, 3.43, 11.08, 0.92	795.4	5.20
Cu L-edge	931.2	2.83	951.1	1.34

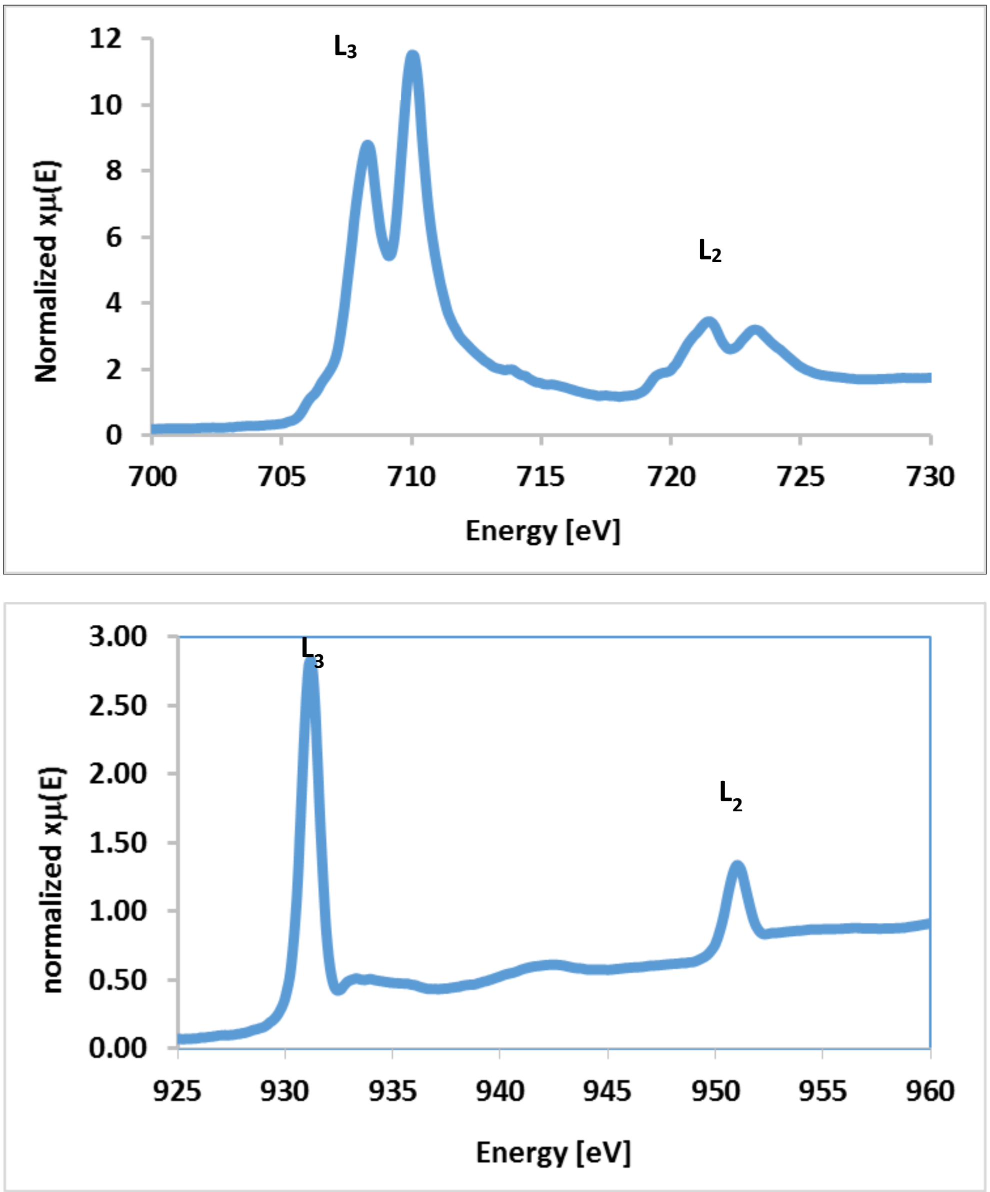

Figure S9. Normalized XANES spectra of iron (top) and copper (bottom) $L_{2,3}$ edges.

Structural analysis of $[Cu_2(AA)_2(ox)]^{2+}$ motifs (AA = bpy or other N-donor ligand)

Some geometrical parameters were discussed as important factors for magnetic couplings [1,2,26]. Hence, we searched CSD for $[(L)Cu(\mu\text{-}ox)Cu(L)]^{2+}$ motif with L being the imine ligand (*e.g.* bpy, phen) and with oxygen ligands in axial positions to maintain the same chromophore (CuN_2O_4) as in the reported compound. The analyzed structure set consists of copper dimers as well as other complexes with such motif; the chains are particularly numerous, but also Cr-Cu-Cu-Cr tetramer was found. Chains are either homometalic composed solely of [Cu(bpy)(ox)] repeating motif or heterometalic with alternately arranged $[Cu_2(bpy)_2(ox)]^{2+}$ cations and anions being polyoxometalates or trisoxalatometalates (see entries in Table S2). It can be seen that: [i] a distorted octahedral environment prevails for copper, [ii] tetragonality [27] of the analyzed compounds ranges from 0.738 to 0.908 but there is no evident relationship between these values and other structural parameters important for magnetic properties. The basal plane of the copper polyhedron was defined by four short Cu-N,O distances. However, if the asymmetry of the Cu-O bonds from bridging oxalate anion is significant, then one short Cu-O bond comes from another O-donor ligand and not from the bridging oxalate. We aim to refer to the strong antiferromagnetic coupling which was detected also in the reported compound (see Magnetic measurements). Therefore, we focus mainly on the entries with short Cu-Cu distances which form two clusters with short separation ranging from 5.096 to 5.180 Å resulting from small asymmetry of Cu-O bonds (Table S2). Analysis of $Cu\text{-}O_{ox}$ bonds showed two clusters: [i] with symmetric bridge and small differences (below 0.04 Å) and [ii] with asymmetric bridge and significant differences exceeding 0.6Å when only one Cu-O bond belongs to copper basal plane (Figure S10ab). It is shown also that in the latter case two opposite Cu-O bonds forming one axis are elongated (*e.g.* D2 and D5 or D3 and D4 in Figure S10c-e. The figure comparing D4-D5 and D2-D3 bonds clearly indicates significant distinctions between entries with long Cu-Cu distances (in red) and with short Cu-Cu separation (in blue). Hence, this parameter is related to the bridge asymmetry and the mutual orientation of oxalate anion and copper basal plane. In **NCU-1**, Cu1 and Cu2 planes are slightly inclined (12.57 °) and they form 3.40 (Cu1) and 9.54 ° (Cu2) with the bisbidentately bridging O71 oxalate anion. It should be noted that in many cases the copper dimer is centrosymmetrical resulting in a parallel orientation of copper basal planes (0.00 °) (Figure S11). Only for 1D ladder motifs they range from 6.28 to 8.25 ° [1,2] and the extreme tilt (26.95 °) was found for chains composed of alternately arranged copper dimers and pseudo-Keggin cores [18]. Therefore, the angle between bridging oxalate and copper basal planes might be more informative. For planes composed of four short Cu-O bonds, usually it is small and only for chains with polyoxometallates [18] it exceeds 10 ° (Table S2). These planes are nearly perpendicularly oriented in compounds with an asymmetric bridges and significantly differing Cu-O bonds (70.94 – 87.45 °). In the reported structure, Cu1 is located almost in the basal plane defined by N_2O_2 environment with shift of 0.004 Å, whereas for Cu2 this distance is substantially bigger (0.112 Å). The Cu(II) shift out of the N_2O_2 plane is usually small and for compounds with symmetric bridge it does not exceed 0.15 Å (Table S2, Figure S12). The bigger deviations occur only for fivefold coordinated copper(II) ions. The auxiliary ligands can affect geometry of the bridge. However, for the majority of these compounds these imine ligands are coplanar (below 5 °) and significant differences are observed only for ladder chains

[12] and chains composed of copper dimers crosslinking pseudo-Keggin cores [18]**Error! Bookmark not defined.** (Figure S13). The biggest tilt (31.92 °) was found in the reported structure with two N1 and N11 bpy molecules from the copper dimer forming two-blade propeller. The shape of the searched motif can be presented by the scatter plot of the distance between bpy centroids *vs*. the angle between those moieties (Figure S14). The smaller distance observed for almost perpendicularly oriented aromatic rings is observed for longer Cu-Cu distances and asymmetric bridge. Hence, we can conclude that in such cases copper ions should be shifted in the same direction and bpy ligands start forming a hemisphere.

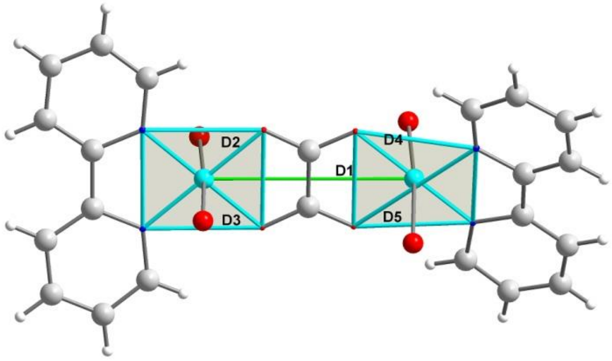

(a)

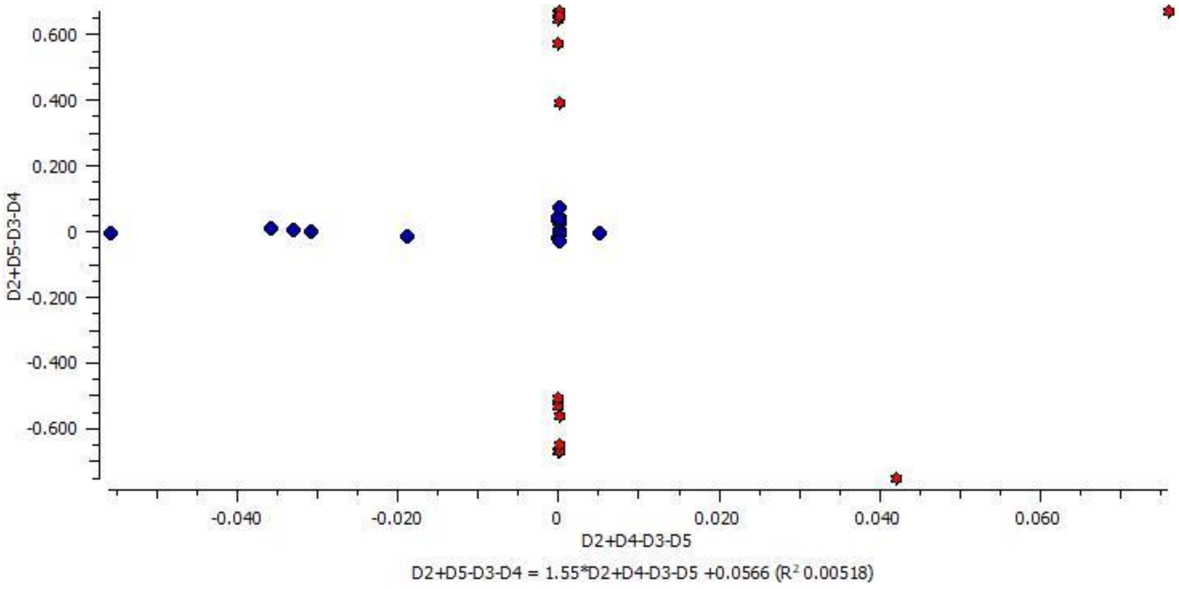

(b)

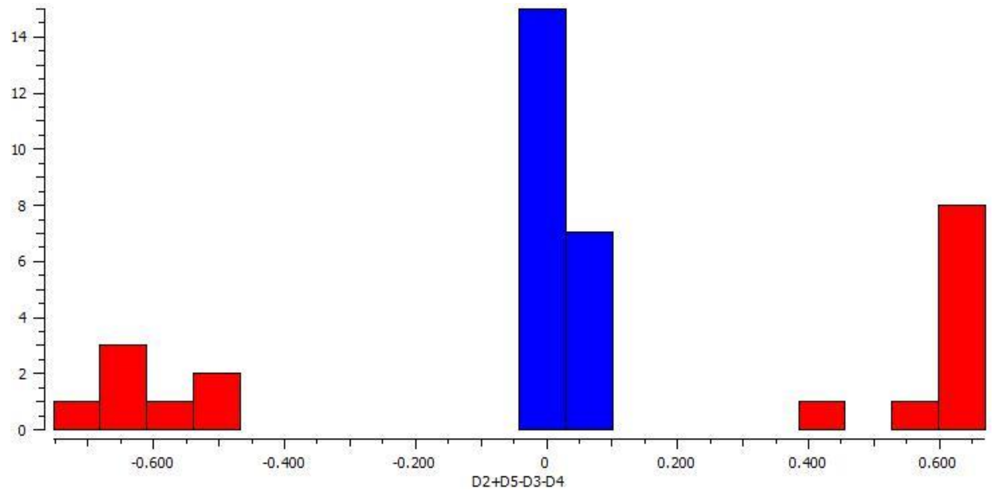

(c)

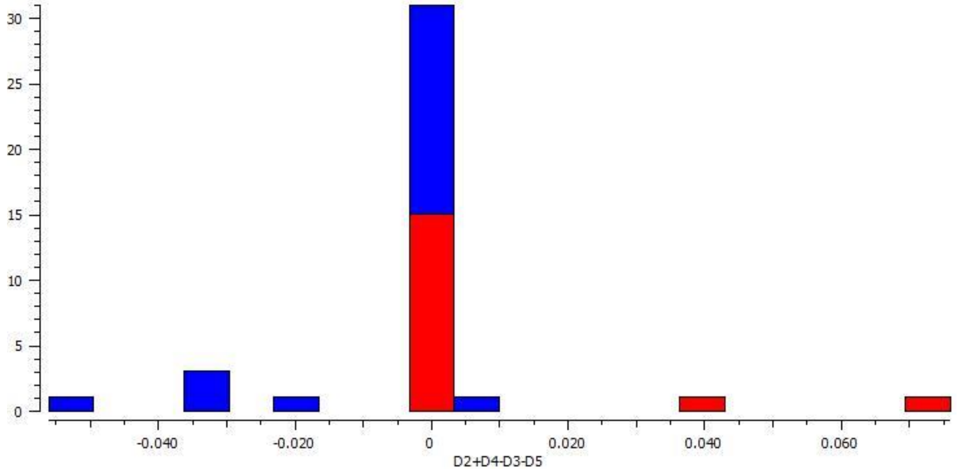

(d)

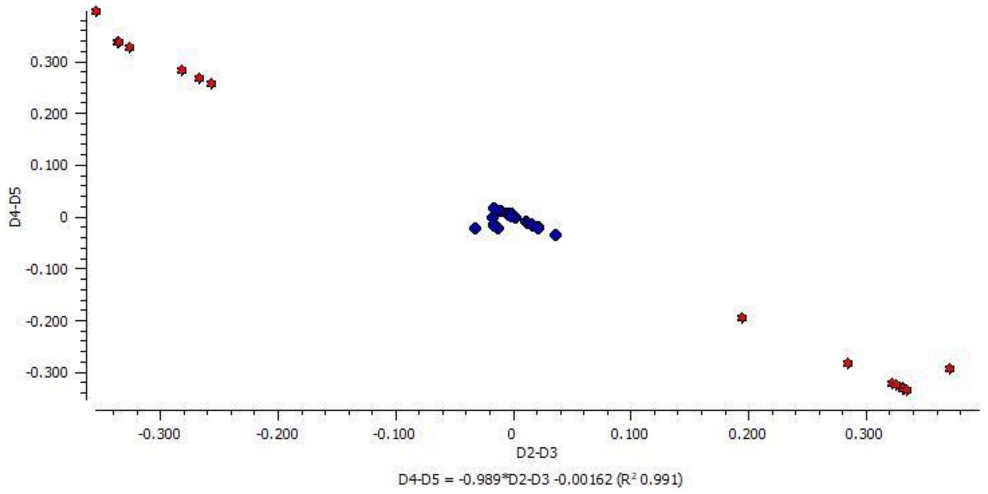

(e)

Figure S10. Parameters describing the oxalate bridge geometry (selected parameters – distances and basal planes – marked in the picture (a)) with entries showing short Cu-Cu distances in blue and with long distances in red. D2, D3, D4 and D5 as well as their relationships are given in [Å].

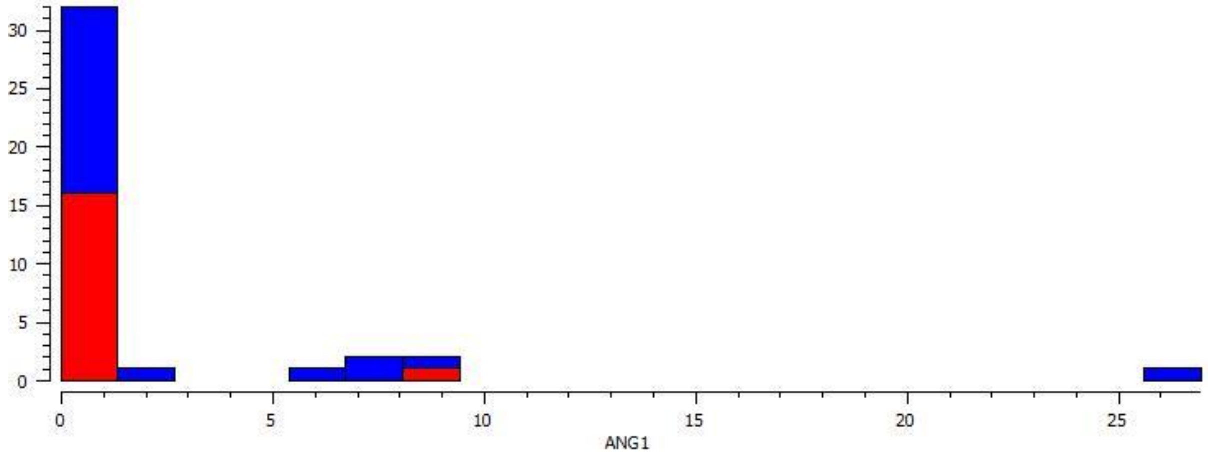

Figure S11. Angle [°] between copper basal planes along the Cu1-ox-Cu2 linkages in the current CSD search (the short Cu-Cu distances in blue and the long ones in red).

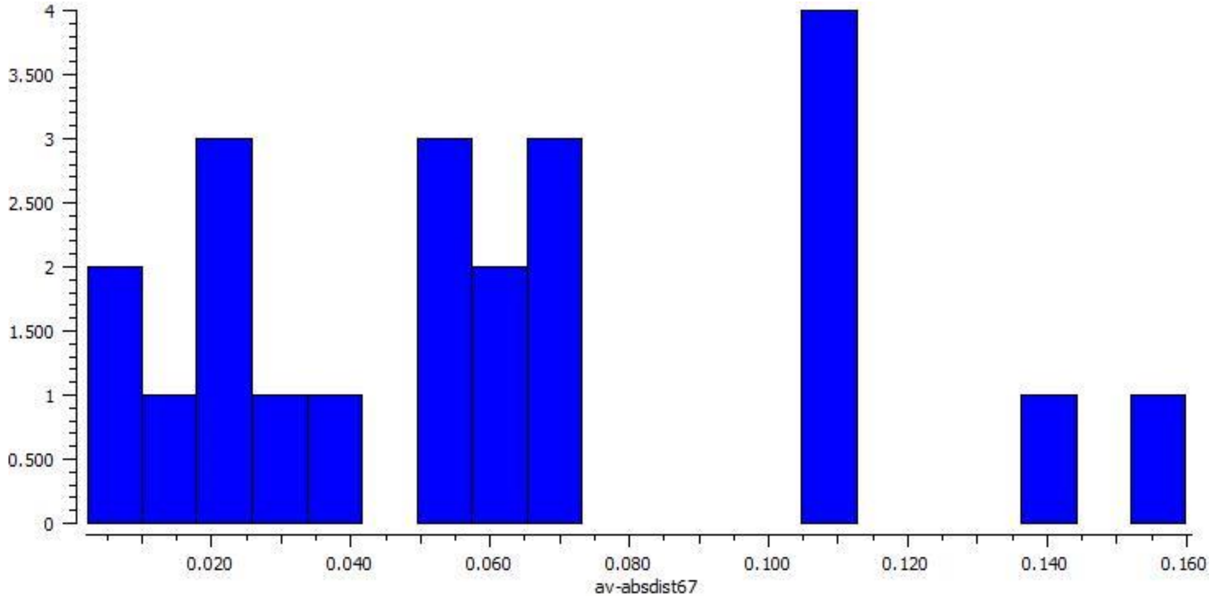

Figure S12. Distribution of Cu atom shift [Å] from the copper base along the Cu1-ox-Cu2 linkages in the current CSD search.

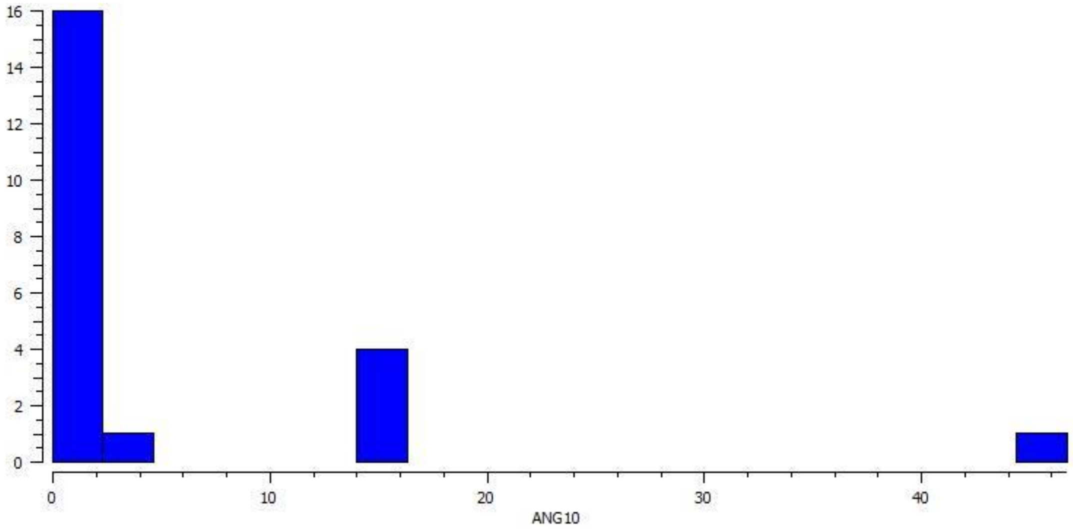

Figure S13. Angles [°] between imine moieties along the Cu1-ox-Cu2 linkages in the current CSD search.

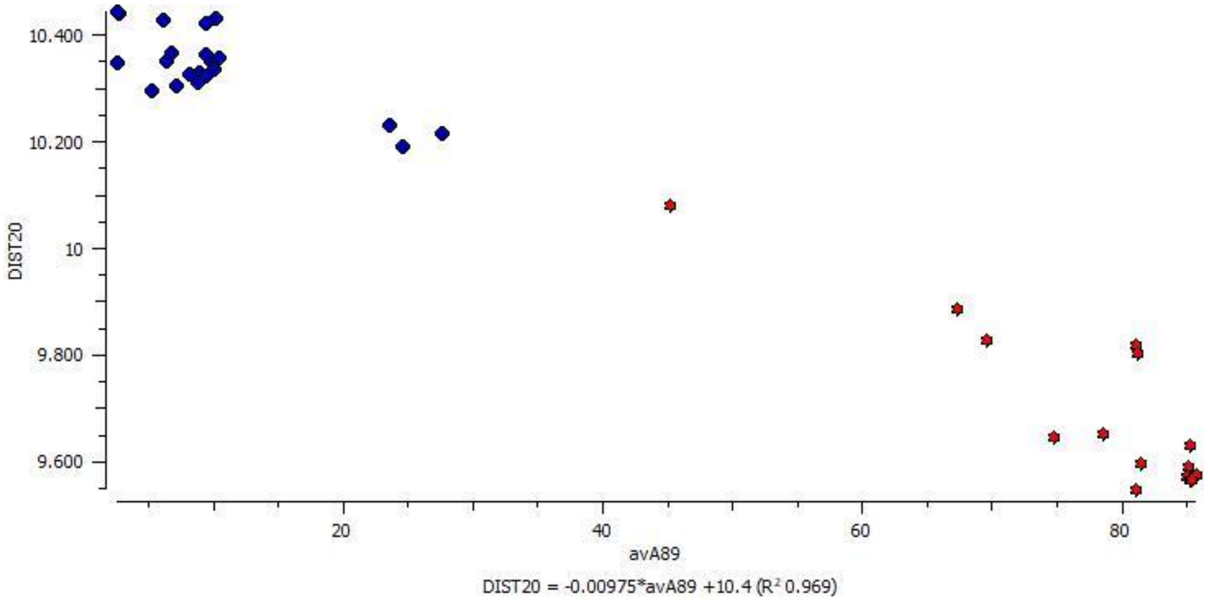

Figure S14. Distance [Å] between bpy centroids *vs*. the average value of an angle [°] between bpy/ox planes for compounds with short distances (the entries with short Cu-Cu distances are given in blue and those with the long ones are given in red).

Computational details of the MF approach

The molecular field equations for this system read

$$M_{A_1 i} = \chi_A [H + \lambda_1 M_{A_2 i} + \lambda_3 M_{Bi+1} + \lambda_5 M_{Bi}], \tag{S1a}$$

$$M_{A_2 i} = \chi_A [H + \lambda_1 M_{A_1 i} + \lambda_2 M_{Bi+1} + \lambda_4 M_{Bi}], \tag{S1b}$$

$$M_{Bi} = \chi_B [H + \lambda_2 M_{A_2 i-1} + \lambda_3 M_{A_1 i-1} + \lambda_4 M_{A_2 i} + \lambda_5 M_{A_1 i}], \tag{S1c}$$

where i=1,…,N, and periodic boundary conditions are satisfied, i.e. $M_{A_1 0} \equiv M_{A_1 N}$, $M_{A_2 0} \equiv M_{A_2 N}$, $M_{BN+1} = M_{B1}$. Quantities χ_A and χ_B denote the magnetic susceptibilities of subsystems A_1, A_2, and B, respectively. Molecular field constants λ_a (a=1,2,…,5) are proportional to the corresponding exchange coupling constants and are given by the formulae

$$\lambda_1 = \frac{J_1}{N_A \mu_B^2 g_A^2}, \quad \lambda_b = \frac{J_b}{N_A \mu_B^2 g_A g_B} \quad (b = 2,3,4,5) \tag{S2}$$

where N_A is the Avogadro number. System of Eqs. (S1a-c) can be solved by algebraic methods yielding

$$M_{A_1 i} = \frac{\chi_A H[1+\chi_A\lambda_1 + \chi_B(\lambda_3+\lambda_5) + \chi_A\chi_B(\lambda_1-\lambda_2+\lambda_3-\lambda_4+\lambda_5)(\lambda_2+\lambda_4)]}{1-\chi_A^2\lambda_1^2 - \chi_A\chi_B[(\lambda_2+\lambda_4)^2+(\lambda_3+\lambda_5)^2] - 2\chi_A^2\chi_B\lambda_1(\lambda_2+\lambda_4)(\lambda_3+\lambda_5)}, \tag{S3a}$$

$$M_{A_2 i} = \frac{\chi_A H[1+\chi_A\lambda_1 + \chi_B(\lambda_2+\lambda_4) + \chi_A\chi_B(\lambda_1+\lambda_2-\lambda_3+\lambda_4-\lambda_5)(\lambda_3+\lambda_5)]}{1-\chi_A^2\lambda_1^2 - \chi_A\chi_B[(\lambda_2+\lambda_4)^2+(\lambda_3+\lambda_5)^2] - 2\chi_A^2\chi_B\lambda_1(\lambda_2+\lambda_4)(\lambda_3+\lambda_5)}, \tag{S3b}$$

$$M_{Bi} = \frac{\chi_B H[1+\chi_A(\lambda_2+\lambda_3+\lambda_4+\lambda_5) - \chi_A^2\lambda_1(\lambda_1-\lambda_2-\lambda_3-\lambda_4-\lambda_5)]}{1-\chi_A^2\lambda_1^2 - \chi_A\chi_B[(\lambda_2+\lambda_4)^2+(\lambda_3+\lambda_5)^2] - 2\chi_A^2\chi_B\lambda_1(\lambda_2+\lambda_4)(\lambda_3+\lambda_5)}, \tag{S3c}$$

independent of index value, i=1,2,…, N. Hence, the molecular field prediction for the total susceptibility of the system calculated per a single A_1A_2B unit reads

$$\chi_{MF} = \frac{1}{N}\sum_{i=1}^{N} \frac{M_{A_1 i} + M_{A_2 i} + M_{Bi}}{H} = \frac{2\chi_A + \chi_B + 2\chi_A\chi_B(\lambda_{24}+\lambda_{35}) + 2\chi_A^2\lambda_1 - \chi_A^2\chi_B[\lambda_1^2 + (\lambda_{24}-\lambda_{35})^2 + 2\lambda_1(\lambda_{24}+\lambda_{35})]}{1-\chi_A^2\lambda_1^2 - \chi_A\chi_B(\lambda_{24}^2+\lambda_{35}^2) - 2\chi_A^2\chi_B\lambda_1\lambda_{24}\lambda_{35}}, \tag{S4}$$

where $\lambda_{24} \equiv \lambda_2 + \lambda_4$ and $\lambda_{35} \equiv \lambda_3 + \lambda_5$. It can be seen that the formula in Eq. (S4) is invariant under the interchange of $\lambda_{24} \leftrightarrow \lambda_{35}$, which is due to the fact that the studied system does not change physically if subject to the interchange of sublattices A_1 and A_2. At sufficiently high temperatures (the actual range depends on the strength of the coupling constants λ_a, a=1,2,…,5) the susceptibilities of the A_1, A_2 and B subsystems can be approximated by the paramagnetic contributions, i.e. $\chi_A = C_A / T$ and $\chi_B = C_B / T$, where $C_A = N_A \mu_B^2 S_A(S_A+1)/3k_B$ and $C_B = N_A \mu_B^2 S_B(S_B+1)/3k_B$ (k_B is the Boltzmann constant). Identifying sublattices A_1 and A_2 with the Cu(II) subsystem (S_{Cu}=1/2), and sublattice B with

the Fe(III) subsystem (S_{Fe}=5/2), and using Eqs. (S2) and (S4) one arrives at the practical formula for the MF molar susceptibility of the studied system $\chi_{MF} = P/Q$, where P and Q are given in Eqs. (6a-b) of the main manuscript.

Definitions of quantities ρ, ρ_α' and ρ_α" (α=0,1)

$$\rho(J_1, J_{23}, J_{45}, \beta) = \frac{a_1(J_1, J_{23}, J_{45}, \beta)}{a_0(J_1, J_{23}, J_{45}, \beta)}$$
$$\rho'_\alpha(J_1, J_{23}, J_{45}, \beta) = \frac{a'_\alpha(J_1, J_{23}, J_{45}, \beta)}{a_0(J_1, J_{23}, J_{45}, \beta)} \quad \text{(S5)}$$
$$\rho''_\alpha(J_1, J_{23}, J_{45}, \beta) = \frac{a''_\alpha(J_1, J_{23}, J_{45}, \beta)}{a_0(J_1, J_{23}, J_{45}, \beta)}$$

$$a_0(J_1, J_{23}, J_{45}, \beta) = 2\left\{\exp\left(-\frac{3}{4}\beta J_1\right) + \exp\left(\frac{1}{4}\beta J_1\right)\left[1 + 2\left(\cosh(\beta\sqrt{S_{Fe}(S_{Fe}+1)}J_{23}) \times \right.\right.\right.$$
$$\times \frac{\sinh(\beta\sqrt{S_{Fe}(S_{Fe}+1)}J_{45})}{\beta\sqrt{S_{Fe}(S_{Fe}+1)}J_{45}} + \cosh(\beta S_{Fe}(S_{Fe}+1)J_{45})\frac{\sinh(\beta\sqrt{S_{Fe}(S_{Fe}+1)}J_{23})}{\beta\sqrt{S_{Fe}(S_{Fe}+1)}J_{23}} - \quad \text{(S6)}$$
$$\left.\left.\left. - \frac{\sinh(\beta\sqrt{S_{Fe}(S_{Fe}+1)}J_{23})}{\beta\sqrt{S_{Fe}(S_{Fe}+1)}J_{23}}\frac{\sinh(\beta\sqrt{S_{Fe}(S_{Fe}+1)}J_{45})}{\beta\sqrt{S_{Fe}(S_{Fe}+1)}J_{45}}\right)\right]\right\}$$

$$a_1(J_1, J_{23}, J_{45}, \beta) = \frac{4\exp\left(\frac{1}{4}\beta J_1\right)}{\beta^2 S_{Fe}(S_{Fe}+1)J_{23}J_{45}}\left[\left(3 + \beta^2 S_{Fe}(S_{Fe}+1)J_{23}^2\right)\cosh(\beta\sqrt{S_{Fe}(S_{Fe}+1)}J_{45}) \times \right.$$
$$\times \frac{\sinh[\beta\sqrt{S_{Fe}(S_{Fe}+1)}J_{23}]}{\beta\sqrt{S_{Fe}(S_{Fe}+1)}J_{23}} + \left(3 + \beta^2 S_{Fe}(S_{Fe}+1)J_{45}^2\right)\cosh(\beta\sqrt{S_{Fe}(S_{Fe}+1)}J_{23}) \times$$
$$\text{(S7)}$$
$$\times \frac{\sinh[\beta\sqrt{S_{Fe}(S_{Fe}+1)}J_{45}]}{\beta\sqrt{S_{Fe}(S_{Fe}+1)}J_{45}} - 3\cosh(\beta\sqrt{S_{Fe}(S_{Fe}+1)}J_{23})\cosh(\beta\sqrt{S_{Fe}(S_{Fe}+1)}J_{45}) -$$
$$\left. - [3 + \beta^2 S_{Fe}(S_{Fe}+1)(J_{23}^2 + J_{45}^2)]\frac{\sinh[\beta\sqrt{S_{Fe}(S_{Fe}+1)}J_{23}]}{\beta\sqrt{S_{Fe}(S_{Fe}+1)}J_{23}}\frac{\sinh[\beta\sqrt{S_{Fe}(S_{Fe}+1)}J_{45}]}{\beta\sqrt{S_{Fe}(S_{Fe}+1)}J_{45}}\right]$$

$$a'_0(J_1, J_{23}, J_{45}, \beta) = 2\exp\left(\frac{1}{4}\beta J_1\right)\frac{\sinh[\beta\sqrt{S_{Fe}(S_{Fe}+1)}J_{23}]}{\beta\sqrt{S_{Fe}(S_{Fe}+1)}J_{23}}\frac{\sinh[\beta\sqrt{S_{Fe}(S_{Fe}+1)}J_{45}]}{\beta\sqrt{S_{Fe}(S_{Fe}+1)}J_{45}} \quad \text{(S8)}$$

$$a'_1(J_1, J_{23}, J_{45}, \beta) = \frac{2\exp\left(\frac{1}{4}\beta J_1\right)}{\beta^2 S_{Fe}(S_{Fe}+1)J_{23}J_{45}}\left[\cosh(\beta\sqrt{S_{Fe}(S_{Fe}+1)}J_{23}) - \frac{\sinh[\beta\sqrt{S_{Fe}(S_{Fe}+1)}J_{23}]}{\beta\sqrt{S_{Fe}(S_{Fe}+1)}J_{23}}\right] \times$$
$$\times \left[\cosh(\beta\sqrt{S_{Fe}(S_{Fe}+1)}J_{45}) - \frac{\sinh[\beta\sqrt{S_{Fe}(S_{Fe}+1)}J_{45}]}{\beta\sqrt{S_{Fe}(S_{Fe}+1)}J_{45}}\right]$$
(S9)

$$a_0''(J_1, J_{23}, J_{45}, \beta) = \frac{\exp\left(\frac{1}{4}\beta J_1\right)}{\beta^2 S_{\mathrm{Fe}}(S_{\mathrm{Fe}}+1)J_{23}J_{45}}\left[\frac{\sinh[\beta\sqrt{S_{\mathrm{Fe}}(S_{\mathrm{Fe}}+1)}(J_{23}+J_{45})]}{\beta\sqrt{S_{\mathrm{Fe}}(S_{\mathrm{Fe}}+1)}(J_{23}+J_{45})} - \frac{\sinh[\beta\sqrt{S_{\mathrm{Fe}}(S_{\mathrm{Fe}}+1)}(J_{23}-J_{45})]}{\beta\sqrt{S_{\mathrm{Fe}}(S_{\mathrm{Fe}}+1)}(J_{23}-J_{45})}\right] \tag{S10}$$

$$a_1''(J_1, J_{23}, J_{45}, \beta) = \frac{\exp\left(\frac{1}{4}\beta J_1\right)}{\beta^2 S_{\mathrm{Fe}}(S_{\mathrm{Fe}}+1)J_{23}J_{45}}\left[\frac{\sinh[\beta\sqrt{S_{\mathrm{Fe}}(S_{\mathrm{Fe}}+1)}(J_{23}+J_{45})]}{\beta\sqrt{S_{\mathrm{Fe}}(S_{\mathrm{Fe}}+1)}(J_{23}+J_{45})} + \frac{\sinh[\beta\sqrt{S_{\mathrm{Fe}}(S_{\mathrm{Fe}}+1)}(J_{23}-J_{45})]}{\beta\sqrt{S_{\mathrm{Fe}}(S_{\mathrm{Fe}}+1)}(J_{23}-J_{45})} - 2\frac{\sinh[\beta\sqrt{S_{\mathrm{Fe}}(S_{\mathrm{Fe}}+1)}J_{23}]}{\beta\sqrt{S_{\mathrm{Fe}}(S_{\mathrm{Fe}}+1)}J_{23}}\frac{\sinh[\beta\sqrt{S_{\mathrm{Fe}}(S_{\mathrm{Fe}}+1)}J_{45}]}{\beta\sqrt{S_{\mathrm{Fe}}(S_{\mathrm{Fe}}+1)}J_{45}}\right] \tag{S11}$$

References

1 L. Kanižaj, L. Androš Dubraja, F. Torić, D. Pajić, K. Molčanov, E. Wenger and M. Jurić, *Inorg. Chem. Front.*, 2019, **6**, 3327-3335.

2 L. Kanižaj, K. Molčanov, F. Torić, D. Pajić, I. Lončarić, A. Šantić and M. Jurić, *Dalton Trans.*, 2019, **48**, 7891-7898.

3 L. Androš Dubraja, M. Jurić, F. Torić and D. Pajić, *Dalton Trans.*, 2017, **46**,11748-11756.

4 M. Julve, A. Gleizes, L. M. Chamoreau, E. Ruiz and M. Verdaguer, *Eur. J. Inorg. Chem.*, 2018, 509–516.

5 H. Oshio and U. Nagashima, *Inorg. Chem.*, 1992, **31**, 3295-3301.

6 O. Castillo, I. Muga, A. Luque, J. M. Gutiérrez-Zorrilla, J. Sertucha, P. Vitoria and P. Román, *Polyhedron*, 1999, **18**, 1235–1245.

7 E. Coronado, M. C. Giménez, C. J. Gómez-García and F. M. Romero, *Polyhedron*, 2003, **22**, 3115–3122.

8 A. Gleizes, M. Julve, M. Verdaguer, J. A. Real,J. Faus, X. Solons, *J. Chem. Soc. Dalton Trans.*, 1992, 3209-3216

9 R. Csonka, J. Kaizer, M. Giorgi, M. Réglier, L. Hajba, J. Mink and G. Speier, *Inorg. Chem.*, 2008, **47**, 6121-6123.

10 D. M. de Faria, M. I. Yoshida, C. B. Pinheiro, K. J. Guedes, K. Krambrock, R. Diniz, L. F. C. de Oliveira and F. C. Machado, *Polyhedron*, 2007, **26**, 4525–4532.

11 J. Luo, M. Hong, Y. Liang and R. Cao, *Acta Cryst.*, 2001, **E57**, m361-m362.

12 B. Xiao, *Z. Kristallogr. NCS*, 2012, **227**, 181-182.

13 Ch. Qin, *Acta Cryst.*, 2007, **E63**, m1006–m1007.

14 A. Castiñeiras, S. Balboa, R. Carballo, J. M. González-Pérez and J. Niclós-Gutiérrez, *Z. Anorg. Allg. Chem.*, 2007, **633**, 717-723.

15 Y. Lü, X. Zhang, X.-B. Cui and J.-Q. Xu, *Inorg. Chem.*, 2018, **57**, 11123−11134.

16 S. Reinoso, P. Vitoria, J. M. Gutiérrez-Zorrilla, L. Lezama, J. M. Madariaga, L. San Felices and A. Iturrospe, *Inorg. Chem.*, 2007, **46**, 4010-4021.

17 S. Reinoso, P. Vitoria, L. Lezama, J. M. Gutiérrez-Zorrilla and A. Luque, *Inorg. Chem.*, 2003, **42**, 3709-3711.
18 L.-N. Xiao, C.-X. Zhao, X.-M. Shi, H. Zhang, W. Wu and X.-B. Cui, *CrystEngComm,* 2018, **20**, 969-977.
19 J. Cao, S. Liu, R. Cao, L. Xie, Y. Ren, Ch. Gao and L. Xu, *Dalton Trans.*, 2008, 115–120.
20 U. García-Couceiro, O. Castillo, A. Luque, J. P. García-Terán, G. Beobide and P. Román, *Cryst. Growth & Des.*, 2006, **6**, 1839-1847.
21 V. G. Vegas, N. Maldonado, O. Castillo, C. J. Gómez-García and P. Amo-Ochoa, *J. Inorg. Biochem.*, 2019, **200**, 110805.
22 M. Julve, M. Verdaguer, A. Gleizes, M. Philoche-Levisalles and O. Kahn, *Inorg. Chem.*, 1984, **23**, 3808-3818.
23 R. K. Hocking, S. DeBeer George, K. N. Raymond, K. O. Hodgson, B. Hedman and E. I. Solomon, *J. Am. Chem. Soc.*, 2010, **132**, 4006–4015.
24 T. M. Muzioł, N. Tereba, R. Podgajny, D. Kędziera and G. Wrzeszcz, *Dalton Trans.*, 2019, **48**, 11536–11546.
25 M. L. Baker, M. W. Mara, J. J. Yan, K. O. Hodgson, B. Hedman and E.I. Solomon, *Coord. Chem. Rev.*, 2017, **345**, 182–208.
26 M. Julve, A. Gleizes, L. M. Chamoreau, E. Ruiz and M. Verdaguer, *Eur. J. Inorg. Chem.*, 2018, 509–516.
27 W. Fitzgerald, J. Foley, D. McSweeney, N. Ray, D. Sheahan, S. Tyagi, B. Hathaway and P. O'Brien, *J.Chem.Soc., Dalton Trans.*, 1982, 1117-1121.
